# Safety and Immunogenicity of a ChAd155-Vectored Respiratory Syncytial Virus Vaccine in Infants 6–7 Months of age: A Phase 1/2 Randomized Trial

**DOI:** 10.1093/infdis/jiad271

**Published:** 2023-07-21

**Authors:** Xavier Sáez-Llorens, Ximena Norero, Marisa Márcia Mussi-Pinhata, Kathia Luciani, Ignacio Salamanca de la Cueva, Javier Díez-Domingo, Eduardo Lopez-Medina, Cristina Epalza, Jerzy Brzostek, Henryk Szymański, François D Boucher, Benhur S Cetin, Tirza De Leon, Ener Cagri Dinleyici, Miguel Ángel Marín Gabriel, Tolga Ince, Mercedes Macias-Parra, Joanne M Langley, Federico Martinón-Torres, Mika Rämet, Ernest Kuchar, Jorge Pinto, Thanyawee Puthanakit, Fernando Baquero-Artigao, Guido Castelli Gattinara, Jose Manuel Merino Arribas, Jose Tomas Ramos Amador, Leszek Szenborn, Bruce Tapiero, Evan J Anderson, James D Campbell, Saul N Faust, Vanja Nikic, Yingjun Zhou, Wenji Pu, Damien Friel, Ilse Dieussaert, Antonio Gonzalez Lopez, Roderick McPhee, Sonia K Stoszek, Nicolas Vanhoutte

**Affiliations:** Department of Infectious Diseases, Hospital del Niño Dr. José Renán Esquivel; Vaccine Research Department, Centro de Vacunación Internacional; Sistema Nacional de Investigación; Secretaria Nacional de Ciencia y Tecnologia, Panama City, Panama; Department of Infectious Diseases, Hospital del Niño Dr. José Renán Esquivel; Vaccine Research Department, Centro de Vacunación Internacional; Department of Pediatrics, Ribeirão Preto Medical School, University of São Paulo, Ribeirão Preto, São Paulo, Brazil; Department of Infectious Diseases, Hospital de Especialidades Pediátricas Omar Torrijos Herrera, Caja de Seguro Social, Panama City, Panama; Unidad de Investigación, Grupo Instituto Hispalense de Pediatria, Sevilla; FISABIO Fundación para el Fomento Investigación Sanitaria y Biomédica de la Comunitat Valenciana, Centro de Investigación Biomédica en Red of Epidemiology and Public Health, Valencia, Spain; Centro de Estudios en Infectología Pediátrica, Department of Pediatrics, Universidad del Valle, Clínica Imbanaco, Grupo Quironsalud, Cali, Colombia; Pediatric Infectious Diseases Unit, Department of Pediatrics, Hospital Universitario 12 de Octubre, Research and Clinical Trials Unit, Instituto de Investigación Sanitaria Hospital 12 de Octubre, Fundación para la Investigación Biomédica del Hospital 12 de Octubre, Madrid, Spain; Oddział Dziecięcy, Zespół Opieki Zdrowotnej w Dębicy, Dębica; Department of Pediatrics, St Hedwig of Silesia Hospital, Trzebnica, Poland; Department of Pediatrics, Centre Hospitalier Universitaire de Québec, Université Laval, Québec, Canada; Department of Pediatric Infectious Diseases, Faculty of Medicine, Erciyes University, Kayseri, Turkey; Department of Vaccines, Cevaxin Sede David, Chiriquí, Panama; Department of Pediatrics, Faculty of Medicine, Eskisehir Osmangazi University, Eskisehir, Turkey; Departamento de Pediatría, Hospital Universitario Puerta de Hierro-Majadahonda, Departamento de Pediatría, Universidad Autónoma de Madrid, Madrid, Spain; Department of Social Pediatrics, Faculty of Medicine, Dokuz Eylul University, Izmir, Turkey; Instituto Nacional de Pediatria, General Director, Mexico City, Mexico; Canadian Center for Vaccinology, Dalhousie University, IWK Health and Nova Scotia Health, Halifax, Canada; Translational Pediatrics and Infectious Diseases Section, Pediatrics Department, Hospital Clínico Universitario de Santiago de Compostela, Santiago de Compostela; Vaccines, Infections and Pediatrics Research Group, Healthcare Research Institute of Santiago de Compostela, Santiago de Compostela; Centro de Investigación Biomédica en Red of Respiratory Diseases, Instituto de Salud Carlos III, Madrid, Spain; Vaccine Research Center, Tampere University, Tampere, Finland; Department of Pediatrics with Clinical Assessment Unit, Medical University of Warsaw, Warsaw, Poland; Department of Pediatrics, School of Medicine, Federal University of Minas Gerais, Belo Horizonte, Brazil; Department of Pediatrics, Faculty of Medicine, Chulalongkorn University, Bangkok, Thailand; Servicio de Pediatría, Enfermedades Infecciosas y Tropicales, Hospital Universitario Infantil La Paz, Centro de Investigación Biomédica en Red de Enfermedades Infecciosas, ISCIII, Madrid, Spain; Centro Vaccinazioni, Dipartimento Pediatrico Universitario Ospedaliero, Istituti di Ricovero e Cura a Carattere Scientifico, Ospedale Pediatrico Bambino Gesù, Lazio, Rome, Italy; Department of Pediatrics, Nuevo Hospital Universitario de Burgos, Burgos; Department of Pediatrics, Universidad Complutense–Instituto de Investigación Sanitaria del Hospital Clínico San Carlos; Centro de Investigación Biomédica en Red de Enfermedades Infecciosas, Madrid, Spain; Department of Pediatrics and Infectious Diseases, Wroclaw Medical University, Wroclaw, Poland; Centre Hospitalier Universitaire Sainte-Justine, Université de Montréal, Montreal, Canada; Departments of Pediatrics and Medicine, Emory University School of Medicine, Atlanta, Georgia; Center for Vaccine Development and Global Health, Department of Pediatrics, University of Maryland School of Medicine, Baltimore, Maryland; National Institute for Health and Care Research Southampton Clinical Research Facility and Biomedical Research Centre, University Hospital Southampton National Health Service Foundation Trust, and Faculty of Medicine and Institute for Life Sciences, University of Southampton, Southampton, United Kingdom; GSK, Biostatistics, Rockville, Maryland; GSK, Biostatistics, Rockville, Maryland; GSK, Biostatistics, Rockville, Maryland; GSK, Vaccines, Wavre, Belgium; GSK, Vaccines, Wavre, Belgium; GSK, Biostatistics, Rockville, Maryland; GSK, Biostatistics, Rockville, Maryland; GSK, Biostatistics, Rockville, Maryland; GSK, Vaccines, Wavre, Belgium

**Keywords:** ChAd155, RSV, immunogenicity, infant, vaccine-associated enhanced respiratory disease

## Abstract

**Background:**

Respiratory syncytial virus (RSV) is a common cause of lower respiratory tract infections in infants. This phase 1/2, observer-blind, randomized, controlled study assessed the safety and immunogenicity of an investigational chimpanzee-derived adenoviral vector RSV vaccine (ChAd155-RSV, expressing RSV F, N, and M2-1) in infants.

**Methods:**

Healthy 6- to 7-month-olds were 1:1:1-randomized to receive 1 low ChAd155-RSV dose (1.5 × 10^10^ viral particles) followed by placebo (RSV_1D); 2 high ChAd155-RSV doses (5 × 10^10^ viral particles) (RSV_2D); or active comparator vaccines/placebo (comparator) on days 1 and 31. Follow-up lasted approximately 2 years.

**Results:**

Two hundred one infants were vaccinated (RSV_1D: 65; RSV_2D: 71; comparator: 65); 159 were RSV-seronaive at baseline. Most solicited and unsolicited adverse events after ChAd155-RSV occurred at similar or lower rates than after active comparators. In infants who developed RSV infection, there was no evidence of vaccine-associated enhanced respiratory disease (VAERD). RSV-A neutralizing titers and RSV F-binding antibody concentrations were higher post–ChAd155-RSV than postcomparator at days 31, 61, and end of RSV season 1 (mean follow-up, 7 months). High-dose ChAd155-RSV induced stronger responses than low-dose, with further increases post–dose 2.

**Conclusions:**

ChAd155-RSV administered to 6- to 7-month-olds had a reactogenicity/safety profile like other childhood vaccines, showed no evidence of VAERD, and induced a humoral immune response.

**Clinical Trials Registration.** NCT03636906.

## Teaser

The investigational adenoviral vector ChAd155-RSV vaccine administered to 6- to 7-month-old infants had a reactogenicity and safety profile like that of other childhood vaccines, showed no evidence of vaccine-associated enhanced respiratory disease, and induced a humoral immune response.

Respiratory syncytial virus (RSV) is a common, contagious pathogen that causes respiratory tract infections (RTIs) in people of all ages. These typically occur in autumn and winter months in temperate regions and in rainy seasons or throughout the year in the tropics [1, 2], although seasonality has been impacted by the coronavirus disease 2019 (COVID-19) pandemic [3–6]. RSV is one of the most common etiologies of lower respiratory tract infections (LRTIs), such as bronchiolitis and pneumonia, in infants and young children [7–10].

Two monoclonal antibodies are available for RSV-LRTI prevention [11, 12], but no pediatric RSV vaccine has been licensed yet. The development of RSV vaccines was slowed down by the failure of an investigational formalin-inactivated vaccine (FI-RSV) in young children >50 years ago [13]. This vaccine did not protect against RSV infection and was associated with severe respiratory illness upon natural RSV infection (particularly in young RSV-naive infants), a phenomenon called vaccine-associated enhanced respiratory disease (VAERD) [13–17]. When evaluating new pediatric RSV vaccine candidates, it is critical to show the absence of VAERD after vaccination of RSV-naive children.

Our study evaluated an investigational RSV vaccine based on a chimpanzee-derived replication-deficient adenoviral vector (ChAd155-RSV). The vector encodes 3 recombinant proteins that are highly conserved among the 2 RSV subtypes (RSV-A and RSV-B): the surface fusion (F) protein (which elicits neutralizing antibodies), the internal nucleocapsid (N) protein, and the transcription antitermination (M2-1) protein (both sources of T-cell epitopes) [13, 18]. In a phase 1 study in 18- to 45-year-old RSV-seropositive adults, ChAd155-RSV had an acceptable safety profile and was immunogenic [19]. In a phase 1/2 study in 12- to 23-month-old RSV-seropositive children, ChAd155-RSV induced neutralizing antibodies and was well tolerated, with no evidence of VAERD upon natural infection [20]. Our study evaluated the safety and immunogenicity of ChAd155-RSV administered to healthy 6- to 7-month-old infants, most of whom were seronaive.

## METHODS

### Study Design and Participants

This phase 1/2, observer-blind, randomized controlled study was performed from 8 April 2019 to 22 July 2021 in 13 countries (Supplementary Table 1). We enrolled healthy, full-term, 6- to 7-month-old infants with no history of confirmed RSV disease or highly compatible clinical manifestations, after obtaining written informed consent from their parents or legally authorized representatives (LARs) (Supplementary Methods).

**Figure 1.**
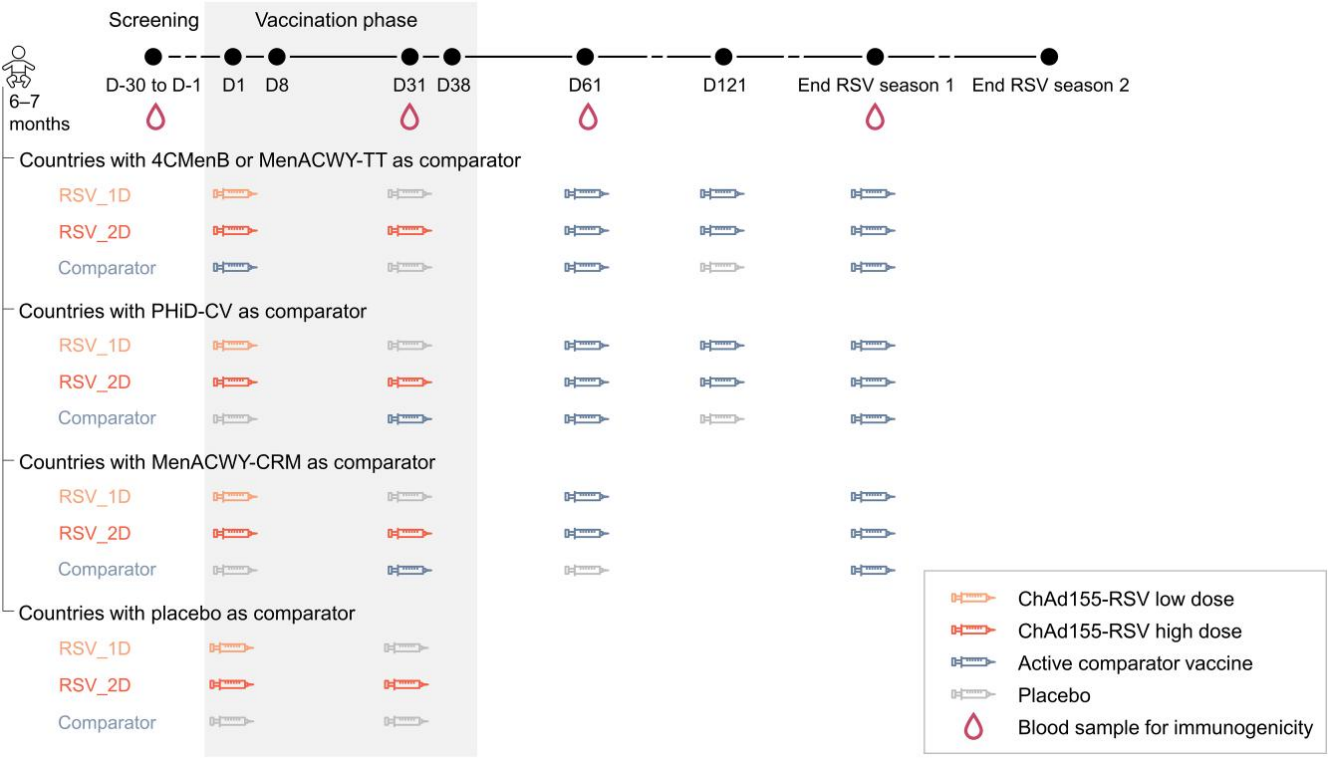
Study design. Abbreviations: 4CMenB, 4-component meningococcal serogroup B vaccine; comparator, group receiving either placebo as dose 1 and 2, or active comparator vaccine as dose 1 or 2 and placebo as the other dose (as indicated)—note, active comparators were given based on approved schedules; D, day; MenACWY-CRM, meningococcal serogroups A, C, W, Y CRM_197_ conjugate vaccine; MenACWY-TT, meningococcal serogroups A, C, W, Y tetanus toxoid conjugate vaccine; PHiD-CV, pneumococcal nontypeable *Haemophilus influenzae* protein D conjugate vaccine; RSV, respiratory syncytial virus; RSV_1D, group receiving 1 low chimpanzee-derived replication-deficient adenoviral vector RSV vaccine (ChAd155-RSV) dose as dose 1 and placebo as dose 2; RSV_2D, group receiving 2 high ChAd155-RSV doses as dose 1 and 2.

Infants were randomized 1:1:1 (Supplementary Methods) to 3 groups: RSV_1D, RSV_2D, or comparator (Figure 1). The RSV_1D group received 1 low ChAd155-RSV dose (1.5 × 10^10^ viral particles) on day 1 and placebo on day 31. The RSV_2D group received 2 high ChAd155-RSV doses (5 × 10^10^ viral particles) on days 1 and 31. The comparator group received either placebo only (days 1 and 31), or placebo and 1 of 4 possible active comparator vaccines depending on the country. Four-component meningococcal serogroup B vaccine (4CMenB, Bexsero, GSK) or meningococcal serogroups A, C, W, Y tetanus toxoid conjugate vaccine (MenACWY-TT, Nimenrix, Pfizer) was given on day 1, followed by placebo on day 31; pneumococcal nontypeable *Haemophilus influenzae* protein D conjugate vaccine (PHiD-CV, Synflorix, GSK) or meningococcal serogroups A, C, W, Y CRM_197_ conjugate vaccine (MenACWY-CRM, Menveo, GSK) were given on day 31, with placebo on day 1. First doses were given before the start of the first RSV season. Infants were followed for approximately 2 years until the end of the second RSV season. RSV seasons were defined based on epidemiological data from before the COVID-19 pandemic (Supplementary Methods). To complete the active comparator vaccine schedules in the comparator groups, children received 4CMenB, MenACWY-TT, or PHiD-CV on day 61 and at the end of the first RSV season (with placebo on day 121), or MenACWY-CRM at the end of the first RSV season (with placebo on day 61). To maintain blinding, children in the ChAd155-RSV groups received 3 doses of 4CMenB, MenACWY-TT, or PHiD-CV or 2 doses of MenACWY-CRM at these same visits (Figure 1). The study was observer-blinded until day 61 and single-blinded thereafter (Supplementary Methods).

The study (ClinicalTrials.gov: NCT03636906) was conducted according to Good Clinical Practice guidelines, the Declaration of Helsinki, and applicable regulatory requirements. The study sites’ institutional review boards or independent ethics committees approved the protocol and its amendments (available on https://www.gsk-studyregister.com/en/trial-details/?id=204894). An internal safety review committee and independent data monitoring committee (IDMC) monitored the participants’ safety.

### Objectives

The primary objective was to evaluate the safety and reactogenicity of 1 or 2 ChAd155-RSV doses until 60 days post–dose 1. Secondary objectives included safety analyses until the end of the second RSV season and evaluation of the occurrence of RSV-RTI and RSV-LRTI until the end of the second RSV season in infants who were considered RSV seronaive at screening (see “Serological Assessments” for a definition of RSV seronaive). Humoral immunogenicity until the end of the first RSV season was also evaluated as a secondary objective. Objectives are detailed in the Supplementary Methods.

### Safety Assessments

The infants’ parents/LARs recorded solicited adverse events (AEs) occurring within 7 days and unsolicited AEs occurring within 30 days postvaccination on diary cards. Serious AEs (SAEs) and AEs leading to study withdrawal were recorded throughout the study. Spontaneous or excessive bleeding was monitored as an AE of specific interest (AESI) (Supplementary Methods). Because of the risk of VAERD with the FI-RSV vaccine [14–17], RSV-LRTIs occurring throughout the study were considered as AESIs. Identification of RSV-LRTIs in the context of AESI reporting was based on the investigators’ clinical judgment considering clinical history, examination, relevant medical evaluation, and results of a locally available diagnostic RSV test. Not all RSV-LRTIs identified by the investigators and reported as AESIs necessarily met the predefined RSV-LRTI case definition used for surveillance (see “RTI Surveillance” and Supplementary Table 2).

### RTI Surveillance

Participants were monitored for RTIs and episodes of difficulty breathing or wheezing through passive and active surveillance contacts by phone or email throughout the study (Supplementary Figure 1). Study staff contacted the infants’ parents/LARs (or designated persons) weekly during the RSV season or monthly outside the RSV season. Parents/LARs/designated persons were reminded to contact the study staff when their children experienced new or worsened RTI symptoms (cough, rhinorrhea, or nasal obstruction) or episodes of difficulty breathing or wheezing. If RTI symptoms developed, an assessment visit was scheduled (Supplementary Figure 1). During this visit, study staff evaluated clinical signs and symptoms of the RTI, measured oxygen saturation and respiratory rate, and collected nasal swabs for RSV-A/RSV-B detection using quantitative reverse-transcription polymerase chain reaction (PCR) (Supplementary Methods). During the RSV season, nasal swabs were also collected monthly from all infants to detect asymptomatic RSV-RTI. To support the safety review of potential VAERD cases, a respiratory viral panel was performed (by multiplex PCR) on all RSV-positive specimens and all LRTIs matching the case definition to evaluate coinfection.

Case definitions for RSV-RTI, RSV-LRTI, and (very) severe LRTI were based on those proposed by the World Health Organization (WHO) (Supplementary Table 2) [21].

### Serological Assessments

Blood samples (2.5 mL) for immunogenicity analyses were drawn from all infants at screening (baseline), day 31, day 61, and at the end of the first RSV season. RSV-A neutralizing titers were measured using a neutralization test with an assay cutoff (lower limit of quantification) of 18 estimated dilution 60 (ED_60_) [22]. RSV F-binding immunoglobulin G (IgG) antibodies were measured using an enzyme-linked immunosorbent assay [22].

For the analyses in RSV-seronaive infants, a baseline RSV-seronaive status was defined as an RSV-A neutralizing titer <63.086 ED_60_ in the baseline sample. This cutoff was determined through modeling of data from an epidemiological study [23] and represents an RSV-A neutralizing titer 2-fold higher than the estimated titer in 6-month-olds due to residual maternal antibodies. Infants with titers equal to or above this value were considered to have experienced an RSV infection.

### Statistical Analysis

The sample size (target: 150 infants; 50 per group) was based on the minimum number of infants needed to detect a VAERD signal with a magnitude like that observed in a study with the FI-RSV vaccine, in which 80% of RSV infections required hospitalization [14] (Supplementary Methods).

For all analyses, data from the different RSV_1D and RSV_2D groups (receiving the various active comparator vaccines/placebo, Figure 1) were pooled into a single RSV_1D and a single RSV_2D group. Data from the comparator groups were either pooled across all active comparators and placebo (comparator group) or across active comparators only (active comparator group, used for the analysis of solicited AEs).

Safety and reactogenicity were analyzed on the exposed set (all infants receiving ≥1 dose of ChAd155-RSV, active comparator, or placebo). The main analyses of RTI and LRTI were done on the exposed set of baseline RSV-seronaive infants. To evaluate the risk of VAERD, we assessed the proportion of baseline RSV-seronaive infants who received ChAd155-RSV, subsequently became infected with RSV, and had a very severe RSV-LRTI (per case definition, Supplementary Table 2). A risk of VAERD like that with the FI-RSV vaccine (for which 80% of RSV infections required hospitalization [14]) was considered unlikely if the upper limit of the 2-sided 90% confidence interval (CI) for this proportion was <80%. In a post hoc analysis, we calculated the relative risk of RSV-LRTI of any severity (per case definition) in a pooled RSV_1D/RSV_2D group compared to the comparator group for baseline RSV-seronaive infants. These analyses were performed on RSV-LRTIs collected until the end of the first RSV season.

The primary immunogenicity analysis was done on the per-protocol population (all vaccinated infants who complied with eligibility criteria and study procedures and had immunogenicity results available). Results focused on here are for baseline RSV-seronaive infants in the per-protocol population. Geometric mean titers (GMTs), geometric mean concentrations, and geometric mean ratios (GMRs) of postvaccination versus baseline titers/concentrations were calculated. For values below the assay cutoffs, values of half the cutoffs were used.

All analyses were descriptive and were done using SAS software version 9.4 (SAS Institute, Cary, North Carolina).

## RESULTS

### Participants

**Figure 2.**
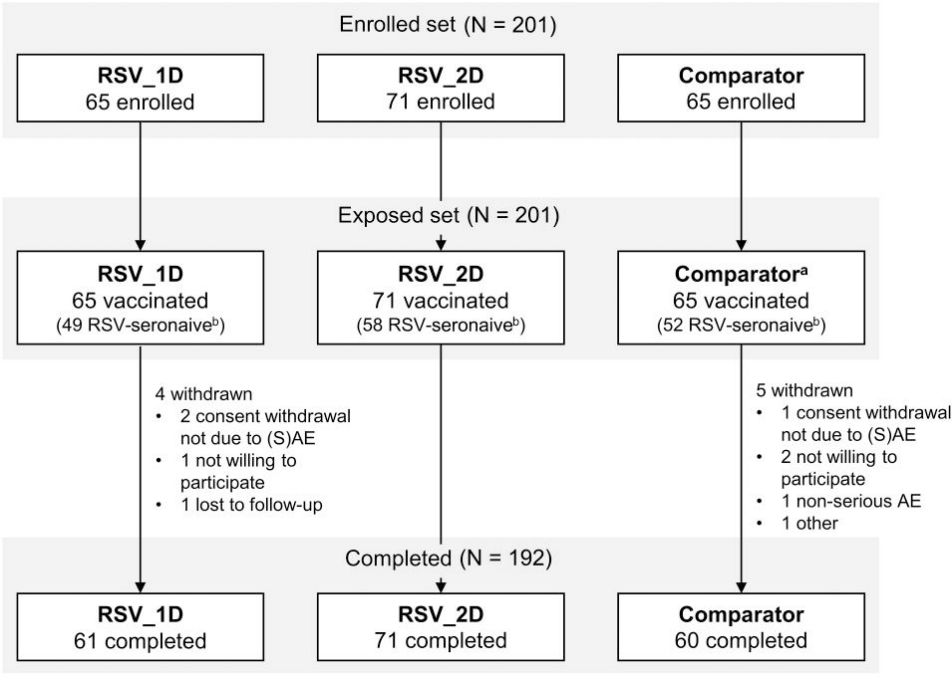
Disposition of participants. ^a^The comparator group included 22 infants who received placebo only and 43 who received active comparator and placebo (29 received 4-component meningococcal serogroup B vaccine, 1 received meningococcal serogroups A, C, W, Y tetanus toxoid conjugate vaccine, 1 received pneumococcal nontypeable *Haemophilus influenzae* protein D conjugate vaccine, and 12 received meningococcal serogroups A, C, W, Y CRM_197_ conjugate vaccine). ^b^RSV-seronaive at screening, ie, RSV-A neutralizing titer <63.086 estimated dilution 60, which represents a titer 2-fold higher than the estimated RSV-A neutralizing titer in infants at 6 months of age due to residual maternal antibodies (based on modeling estimates). Abbreviations: comparator, group receiving either placebo as dose 1 and 2, or active comparator vaccine as dose 1 or 2 and placebo as the other dose (pooled); RSV, respiratory syncytial virus; RSV_1D, group receiving 1 low chimpanzee-derived replication-deficient adenoviral vector RSV vaccine (ChAd155-RSV) dose as dose 1 and placebo as dose 2; RSV_2D, group receiving 2 high ChAd155-RSV doses as dose 1 and 2; (S)AE, (serious) adverse event.

In total, 201 infants were enrolled and vaccinated (RSV_1D: 65; RSV_2D: 71; comparator: 65); 192 (95.5%) completed the study (Figure 2). All infants in the RSV_2D group received a second ChAd155-RSV dose. Of all infants, 159 were baseline RSV-seronaive (RSV_1D: 49; RSV_2D: 58; comparator: 52). The mean follow-up time was approximately 7 months until the end of RSV season 1 and approximately 19 months until the end of season 2. Demographic characteristics were balanced between groups (Table 1).

**Table 1.** Baseline Characteristics of Participants (Exposed Set)

Characteristic	RSV_1D	RSV_2D	Comparator	Total
	(n = 65)	(n = 71)	(n = 65)	(N = 201)
Age at dose 1, mo				
Mean ± SD	6.4 ± 0.5	6.5 ± 0.5	6.5 ± 0.5	6.5 ± 0.5
Median (range)	6.0 (6–7)	7.0 (6–7)	7.0 (6–7)	6.0 (6–7)
Sex, No. (%)				
Female	32 (49.2)	33 (46.5)	31 (47.7)	96 (47.8)
Male	33 (50.8)	38 (53.5)	34 (52.3)	105 (52.2)
Race/ethnicity, No. (%)				
American Indian/Alaska Native	1 (1.5)	1 (1.4)	2 (3.1)	4 (2.0)
Asian	1 (1.5)	1 (1.4)	1 (1.5)	3 (1.5)
Black/African American	0 (0.0)	1 (1.4)	0 (0.0)	1 (0.5)
White	38 (58.5)	39 (54.9)	37 (56.9)	114 (56.7)
Other^a^	25 (38.5)	29 (40.8)	25 (38.5)	79 (39.3)

Abbreviations: comparator, group receiving either placebo as dose 1 and 2, or active comparator vaccine as dose 1 or 2 and placebo as the other dose (pooled); No. (%), number (percentage) of participants in the specified category; RSV, respiratory syncytial virus; RSV_1D, group receiving 1 low chimpanzee-derived replication-deficient adenoviral vector RSV vaccine (ChAd155-RSV) dose as dose 1 and placebo as dose 2; RSV_2D, group receiving 2 high ChAd155-RSV doses as dose 1 and 2; SD, standard deviation.

^a^Most infants in this category (68/79) were reported as “Mixed” without further information. The remaining were reported as “Mexican heritage,” “Latin American,” “Mestizo,” “Mulatto,” and “White and Asian.”

### Reactogenicity and Safety

**Figure 3.**
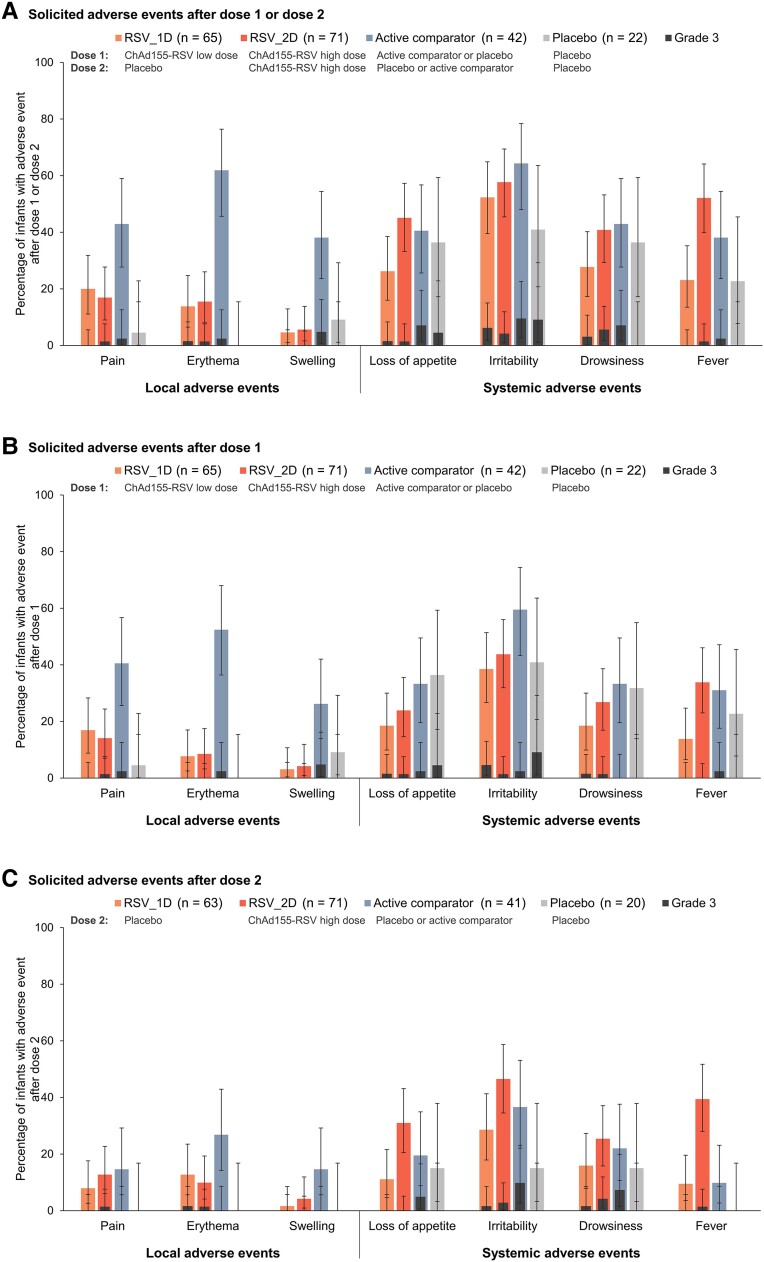
Solicited adverse events within 7 days after vaccination (exposed set). Error bars depict 95% confidence intervals. Graphs show the percentage of infants with solicited adverse events after at least 1 of the 2 doses (*A*), after dose 1 (*B*), and after dose 2 (*C*). Grade 3 was defined as follows: crying when the limb was moved/limb was spontaneously painful for pain; diameter >20 mm for erythema and swelling; not eating at all for loss of appetite; crying inconsolably/preventing normal activities for irritability; preventing normal activities for drowsiness; temperature >40°C for fever. Abbreviations: active comparator, group receiving active comparator vaccine as dose 1 or 2 and placebo as the other dose; n, total number of participants with available results; placebo, group receiving placebo as dose 1 and 2; RSV, respiratory syncytial virus; RSV_1D, group receiving 1 low chimpanzee-derived replication-deficient adenoviral vector RSV vaccine (ChAd155-RSV) dose as dose 1 and placebo as dose 2; RSV_2D, group receiving 2 high ChAd155-RSV doses as dose 1 and 2.

Administration site pain and erythema were the most frequent solicited local AEs, with pain reported in 20.0% (RSV_1D), 16.9% (RSV_2D), 42.9% (active comparator), and 4.5% (placebo) and erythema in 13.8% (RSV_1D), 15.5% (RSV_2D), 61.9% (active comparator), and 0.0% (placebo) of infants after any dose (Figure 3, Supplementary Table 3). Solicited local AEs were more commonly or similarly reported after vaccination with ChAd155-RSV than with placebo, but less commonly than with the active comparators. Grade 3 local AEs occurred in ≤1.5% of ChAd155-RSV, ≤4.8% of active comparator, and 0.0% of placebo recipients (Figure 3, Supplementary Table 3). Irritability was the most common solicited systemic AE, reported in 52.3% (RSV_1D), 57.7% (RSV_2D), 64.3% (active comparator), and 40.9% (placebo) of infants after any dose (Figure 3, Supplementary Table 3). Systemic AEs were reported with similar rates in all groups, except for fever, which occurred more frequently in the RSV_2D (52.1%) than in the RSV_1D group (23.1%) (Figure 3, Supplementary Table 3). The rate of fever in the RSV-2D group after both dose 1 (33.8%) and dose 2 (39.4%) was similar to that in infants receiving 4CMenB as comparator (39.3%) (Supplementary Figure 2). Grade 3 solicited systemic AEs occurred in ≤6.2% of ChAd155-RSV and ≤9.5% of active comparator and placebo recipients (Figure 3, Supplementary Table 3). Most solicited AEs resolved within 1–4 days postvaccination. No increase in reactogenicity was seen after the second versus the first ChAd155-RSV dose (Figure 3, Supplementary Table 3).

Unsolicited AEs were reported in 52.3% (95% CI, 39.5%–64.9%), 63.4% (95% CI, 51.1%–74.5%), and 55.4% (95% CI, 42.5%–67.7%) of RSV_1D, RSV_2D, and comparator recipients, respectively, and grade 3 unsolicited AEs in 6.2% (95% CI, 1.7%–15.0%), 1.4% (95% CI, .0–7.6%), and 3.1% (95% CI, .4%–10.7%). The most common were infections and gastrointestinal disorders. Unsolicited AEs assessed as vaccination-related by the investigator occurred in 6.2% (95% CI, 1.7%–15.0%), 11.3% (95% CI, 5.0%–21.0%), and 7.7% (95% CI, 2.5%–17.0%) of infants in the 3 groups, respectively. One of these (gastrointestinal hemorrhage in RSV-1D, see below) was of grade 3 intensity.

Throughout the study, 7 (10.8% [95% CI, 4.4%–20.9%]) infants in RSV_1D, 11 (15.5% [95% CI, 8.0%–26.0%]) in RSV_2D, and 3 (4.6% [95% CI, 1.0%–12.9%]) in the comparator group experienced nonfatal SAEs; most were infections and were considered unrelated to vaccine/placebo (Supplementary Table 4). Of these, 3 (RSV_1D), 3 (RSV-2D), and 1 (comparator) SAEs occurred within 30 days after first or second vaccination (Supplementary Table 5). Two SAEs were considered as possibly ChAd155-RSV related by the investigator. One infant (RSV_2D) had type 1 diabetic ketoacidosis starting 357 days post–dose 2 and ongoing at study end. Given the time-to-onset and an alternate infectious/inflammatory etiology contributing to the presentation of diabetes, the sponsor did not consider this event ChAd155-RSV related (Supplementary Table 4). One infant (RSV_1D) had gastrointestinal hemorrhage 16 days post–dose 1, which resolved after 10 days. Given a probable infectious etiology, the presence of anal fissure, and increased platelet count, the sponsor did not consider this event ChAd155-RSV related (Supplementary Table 4).

Among baseline RSV-seronaive participants, RSV-LRTIs were reported as AESIs in 5 (10.2% [95% CI, 3.4%–22.2%]), 6 (95% CI, 10.3% [3.9%–21.2%]), and 5 (95% CI, 9.6% [3.2%–21.0%]) participants in the RSV_1D, RSV_2D, and comparator groups, respectively, throughout the study.

### RSV-RTI Surveillance

Among baseline RSV-seronaive participants, 40.8% (RSV_1D), 29.3% (RSV_2D), and 48.1% (comparator) had an RSV infection; 34.7% (RSV_1D), 25.9% (RSV_2D), and 44.2% (comparator) had an RSV-RTI; and 6.1% (RSV_1D), 5.2% (RSV_2D), and 7.7% (comparator) had an RSV-LRTI (based on WHO case definitions) during the study (Table 2). Nearly all RSV infections occurred in the first RSV season. Similar results were observed in infants in the exposed set (which included baseline RSV-seronaive and non-seronaive infants) (Supplementary Table 6).

**Table 2.** Respiratory Syncytial Virus (RSV) Surveillance From Dose 1 Until the End of the Second RSV Season (Exposed Set of Baseline RSV-Seronaive Infants)

Category	RSV_1D		RSV_2D		Comparator	
	(n = 49)		(n = 58)		(n = 52)	
	No.	% (95% CI)	No.	% (95% CI)	No.	% (95% CI)
No infection	29	59.2 (44.2–73.0)	41	70.7 (57.3–81.9)	27	51.9 (37.6–66.0)
RSV infection (symptomatic or asymptomatic)^a^	20	40.8 (27.0–55.8)	17	29.3 (18.1–42.7)	25	48.1 (34.0–62.4)
RSV-RTI^b^	17	34.7 (21.7–49.6)	15	25.9 (15.3–39.0)	23	44.2 (30.5–58.7)
RSV-LRTI	3	6.1 (1.3–16.9)	3	5.2 (1.1–14.4)	4	7.7 (2.1–18.5)
Severe RSV-LRTI	1	2.0 (.1–10.9)	1	1.7 (.0–9.2)	3	5.8 (1.2–15.9)
Very severe RSV-LRTI	0	0.0 (.0–7.3)	0	0.0 (.0–6.2)	0	0.0 (.0–6.8)
All-cause LRTI	13	26.5 (14.9–41.1)	11	19.0 (9.9–31.4)	8	15.4 (6.9–28.1)
RSV hospitalization^c^	1	2.0 (.1–10.9)	1	1.7 (.0–9.2)	1	1.9 (.0–10.3)
RSV-LRTI hospitalization^c^	1	2.0 (.1–10.9)	0	0.0 (.0–6.2)	1	1.9 (.0–10.3)
Severe RSV-LRTI hospitalization^c^	1	2.0 (.1–10.9)	0	0.0 (.0–6.2)	1	1.9 (.0–10.3)
Very severe RSV-LRTI hospitalization^c^	0	0.0 (.0–7.3)	0	0.0 (.0–6.2)	0	0.0 (.0–6.8)
All-cause LRTI hospitalization	2	4.1 (.5–14.0)	1	1.7 (.0–9.2)	1	1.9 (.0–10.3)

The n values indicate the total number of participants in the exposed set who were RSV-seronaive at baseline, ie, had an RSV-A neutralizing titer <63.086 estimated dilution 60. The No. and % indicate number/percentage of participants meeting the specified case definition at least once (case definitions were based on those proposed by the World Health Organization; see Supplementary Table 2).

Abbreviations: CI, confidence interval; LRTI, lower respiratory tract infection; RSV_1D, group receiving 1 low chimpanzee-derived replication-deficient adenoviral vector RSV vaccine (ChAd155-RSV) dose as dose 1 and placebo as dose 2; RSV_2D, group receiving 2 high ChAd155-RSV doses as dose 1 and 2; comparator, group receiving either placebo as dose 1 and 2, or active comparator vaccine as dose 1 or 2 and placebo as the other dose (pooled); RTI, respiratory tract infection.

^a^All confirmed RSV infections based on central reverse-transcription polymerase chain reaction testing.

^b^This included both upper and lower respiratory tract infections.

^c^Confirmed RSV infection and hospitalized for acute medical condition.

None of the RSV infections progressed to very severe RSV-LRTI. The 90% CI for the risk that ChAd155-RSV induced very severe LRTI was 0.0–14.6% in RSV_1D and 0.0–17.1% in RSV_2D recipients (based on 19 RSV infections in RSV_1D and 16 in RSV_2D at the end of RSV season 1). Additionally, the relative risk of RSV-LRTI in the pooled ChAd155-RSV versus the comparator group was 0.73 (95% CI, .22–2.47) at the end of RSV season 1.

### Immunogenicity

**Figure 4.**
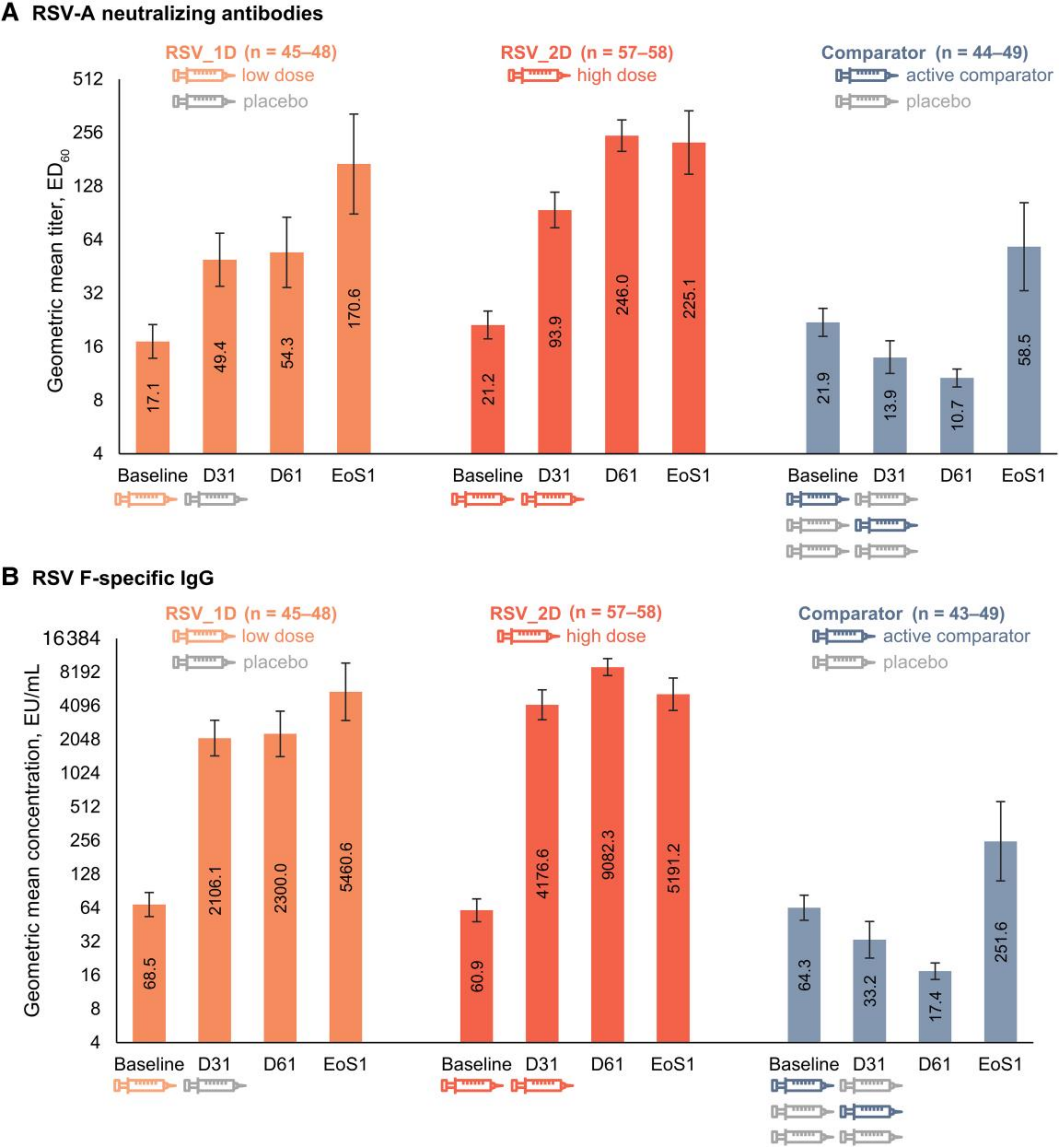
Respiratory syncytial virus (RSV)–A neutralizing geometric mean titers (*A*) and RSV F-binding immunoglobulin G geometric mean concentrations (*B*) (per-protocol population of baseline RSV-seronaive infants). Error bars depict 95% confidence intervals. Abbreviations: comparator, group receiving either placebo as dose 1 and 2, or active comparator vaccine as dose 1 or 2 and placebo as the other dose (pooled); D31, 30 days postvaccination; D61, 60 days postvaccination; ED_60_, estimated dilution 60; EoS1, end of respiratory syncytial virus season 1; EU, enzyme-linked immunosorbent assay units; IgG, immunoglobulin G; n, total number of participants with available results at the indicated timepoint; RSV-A, respiratory syncytial virus subtype A; RSV_1D, group receiving 1 low chimpanzee-derived replication-deficient adenoviral vector RSV vaccine (ChAd155-RSV) dose as dose 1 and placebo as dose 2; RSV_2D, group receiving 2 high ChAd155-RSV doses as dose 1 and 2; RSV F, respiratory syncytial virus fusion protein.

The per-protocol population included 191 infants, of whom 152 were RSV-seronaive at baseline. Among RSV-seronaive infants, RSV-A neutralizing GMTs at days 31, 61, and at the end of RSV season 1 were markedly higher in both ChAd155-RSV groups than in the comparator group (Table 3, Figure 4). One dose of the high-dose formulation induced a greater RSV-A neutralizing response than the low-dose formulation (GMRs of 4.44 [RSV_2D] and 2.88 [RSV_1D] at day 31 vs baseline; Table 3). The second ChAd155-RSV dose further increased the RSV-A neutralizing GMTs at day 61, with a GMR (vs baseline) of 11.80 (Table 3, Figure 4). RSV-A neutralizing GMTs were higher at the end of RSV season 1 than at day 61 in the RSV_1D and comparator groups and similar at these 2 timepoints in the RSV_2D group (Table 3, Figure 4).

**Table 3.** Respiratory Syncytial Virus (RSV)–A Neutralizing Titers and RSV F–Binding Immunoglobulin G Concentrations (Per-Protocol Population of Baseline RSV-Seronaive Infants)

Timepoint		RSV_1D		RSV_2D		Comparator
RSV-A neutralizing titers						
% ≥ assay cutoff	No.	% (95% CI)	No.	% (95% CI)	No.	% (95% CI)
Baseline	48	45.8 (31.4–60.8)	58	67.2 (53.7–79.0)	49	71.4 (56.7–83.4)
Day 31	48	87.5 (74.8–95.3)	57	100 (93.7–100)	49	36.7 (23.4–51.7)
Day 61	47	85.1 (71.7–93.8)	57	100 (93.7–100)	44	18.2 (8.2–32.7)
End RSV season 1	45	77.8 (62.9–88.8)	58	100 (93.8–100)	49	53.1 (38.3–67.5)
GMT	No.	GMT (95% CI), ED_60_	No.	GMT (95% CI), ED_60_	No.	GMT (95% CI), ED_60_
Baseline	48	17.1 (13.8–21.3)	58	21.2 (17.7–25.4)	49	21.9 (18.3–26.3)
Day 31	48	49.4 (35.0–69.7)	57	93.9 (74.6–118.3)	49	13.9 (11.3–17.3)
Day 61	47	54.3 (34.4–85.7)	57	246.0 (200.9–301.2)	44	10.7 (9.5–12.0)
End RSV season 1	45	170.6 (89.3–326.0)	58	225.1 (149.3–339.2)	49	58.5 (33.1–103.3)
GMR	No.	GMR (95% CI)	No.	GMR (95% CI)	No.	GMR (95% CI)
Day 31/baseline	48	2.88 (1.89–4.39)	57	4.44 (3.32–5.92)	49	0.64 (.51–.79)
Day 61/baseline	47	3.12 (1.90–5.13)	57	11.80 (8.80–15.82)	44	0.50 (.41–.60)
End RSV season 1/baseline	45	10.25 (5.16–20.36)	58	10.61 (6.89–16.36)	49	2.67 (1.46–4.88)
RSV F-binding IgG (ELISA)						
% ≥ assay cutoff	No.	% (95% CI)	No.	% (95% CI)	No.	% (95% CI)
Baseline	48	93.8 (82.8–98.7)	58	86.2 (74.6–93.9)	49	87.8 (75.2–95.4)
Day 31	48	100 (92.6–100)	58	98.3 (90.8–100)	48	56.3 (41.2–70.5)
Day 61	46	100 (92.3–100)	57	100 (93.7–100)	43	30.2 (17.2–46.1)
End RSV season 1	45	100 (92.1–100)	58	100 (93.8–100)	48	58.3 (43.2–72.4)
GMC	No.	GMC (95% CI), EU/mL	No.	GMC (95% CI), EU/mL	No.	GMC (95% CI), EU/mL
Baseline	48	68.5 (53.6–87.7)	58	60.9 (48.2–76.9)	49	64.3 (49.7–83.0)
Day 31	48	2106.1 (1460.9–3036.2)	58	4176.6 (3066.9–5687.9)	48	33.2 (22.8–48.3)
Day 61	46	2300.0 (1441.2–3670.4)	57	9082.3 (7635.2–10 803.6)	43	17.4 (14.7–20.6)
End RSV season 1	45	5460.6 (3023.2–9863.4)	58	5191.2 (3719.1–7246.1)	48	251.6 (111.1–569.9)
GMR	No.	GMR (95% CI)	No.	GMR (95% CI)	No.	GMR (95% CI)
Day 31/baseline	48	30.72 (19.41–48.64)	58	68.62 (45.53–103.41)	48	0.51 (.39–.67)
Day 61/baseline	46	32.90 (19.11–56.64)	57	150.00 (107.17–209.95)	43	0.27 (.22–.33)
End RSV season 1/baseline	45	83.58 (42.02–166.25)	58	85.29 (58.97–123.35)	48	3.94 (1.69–9.21)

No. indicates total number of participants in the per-protocol population who were RSV seronaive at baseline (ie, had an RSV-A neutralizing titer <63.086 ED_60_) and had available results at the indicated timepoint (for GMRs, at the indicated postvaccination timepoint and at baseline).

Abbreviations: % ≥ assay cutoff, percentage of participants with an RSV-A neutralizing titer ≥18 ED_60_ (lower limit of quantification) or an RSV F-binding IgG antibody concentration ≥25 EU/mL (limit of detection) measured by enzyme-linked immunosorbent assay; CI, confidence interval; ED_60_, estimated dilution 60; ELISA, enzyme-linked immunosorbent assay; EU, enzyme-linked immunosorbent assay units; GMC, geometric mean concentration; GMR, geometric mean of individual ratios of titers/concentrations at the indicated postvaccination timepoint over baseline; GMT, geometric mean titer; IgG, immunoglobulin G; RSV-A, RSV subtype A; RSV_1D, group receiving 1 low chimpanzee-derived replication-deficient adenoviral vector RSV vaccine (ChAd155-RSV) dose as dose 1 and placebo as dose 2; RSV_2D, group receiving 2 high ChAd155-RSV doses as dose 1 and 2; comparator, group receiving either placebo as dose 1 and 2, or active comparator vaccine as dose 1 or 2 and placebo as the other dose (pooled); RSV F, RSV fusion protein.

RSV F-binding IgG responses followed comparable trends (Table 3, Figure 4).

Similar results were obtained in the per-protocol population (including both RSV-seronaive and non-seronaive infants) (Supplementary Table 7).

## DISCUSSION

This randomized phase 1/2 study showed that administration of the investigational ChAd155-RSV vaccine to 6- to 7-month-old, mostly RSV-seronaive infants had a reactogenicity and safety profile like that of other childhood vaccines, with no indication of VAERD, and induced a humoral immune response. Our findings were in line with those of the first-in-human study with ChAd155-RSV in adults [19] and the phase 1/2 study in 12- to 23-month-old RSV-seropositive children [20].

Most solicited AEs after ChAd155-RSV vaccination were transient, of mild or moderate intensity, and were reported at similar or lower rates than in the active comparator group. Solicited AEs occurred at comparable rates in the 2 ChAd155-RSV groups, except for fever, which was more common in the RSV_2D than the RSV_1D group but occurred at a similar rate as after 4CMenB vaccination, which was the most pyrogenic comparator. The reported unsolicited AE rates were similar across study groups, and the types of AEs were consistent with usual illnesses occurring in infants. One case of gastrointestinal hemorrhage occurred, which was judged by the investigator as possibly ChAd155-RSV related. The IDMC raised no safety concerns, and the sponsor considered the event as unrelated to vaccination based on clinical and laboratory evidence and a more probable alternative etiology. SAEs occurred more frequently in the ChAd155-RSV groups (10.8% and 15.5%) than the comparator group (4.6%). The difference was mostly due to a higher rate of infections in the ChAd155-RSV groups, but no common diagnosis or etiology could be found among these infections. Interpretation of this imbalance is limited by the study's small sample size.

There was no evidence to suggest that ChAd155-RSV vaccination in RSV-seronaive 6- to 7-month-old infants induced VAERD after natural RSV infection. None of the RSV infections in ChAd155-RSV–vaccinated seronaive participants progressed to very severe RSV-LRTI, and the relative risk (0.73) of RSV-LRTI in the pooled ChAd155-RSV group versus the comparator group suggests that an increase in RSV-LRTI in vaccinated infants was unlikely (although the CI around the relative risk was wide). The mechanisms that lead to VAERD after FI-RSV vaccination are not fully understood but may involve suboptimal humoral immune responses, absence of cellular cytotoxic responses, and a Th2-biased cellular response [24, 25]. Chimpanzee-derived adenoviral vector-based vaccines induce a humoral and cellular immune response [26], which would reduce the risk of VAERD. Preclinical studies in animal models showed that ChAd155-RSV was immunogenic and efficacious, with no signs of VAERD after RSV challenge.

A single vaccination with both the low-dose and high-dose ChAd155-RSV formulations increased RSV-A neutralizing titers and RSV F-binding IgG levels. The high-dose formulation elicited a stronger response than the low-dose formulation, and the second ChAd155-RSV dose induced a further increase in antibody levels. Antibody levels in both ChAd155-RSV groups remained above baseline levels and above levels in the comparator group at the end of the first RSV season. Natural RSV infection over the course of the season appeared to boost immunity, as evident from increased antibody levels observed in the RSV_1D and comparator groups.

The rates of RSV infection, RSV-RTI, and RSV-LRTI were numerically lowest in the RSV_2D group and highest in the comparator group over the 2 RSV seasons. Nevertheless, vaccine impact on RSV illness seemed low compared to that of the monoclonal antibody nirsevimab [27] and a maternal RSV-prefusion F candidate vaccine [28, 29]. However, our study was not powered (sample size too low) to draw conclusions on ChAd155-RSV vaccine efficacy. ChAd155-RSV expresses a wild-type version of the F protein with a deletion of the transmembrane domain. Experiments in cell culture indicated that the protein expressed by ChAd155-RSV is present in both the prefusion and postfusion conformation (unpublished data). Prefusion F has been shown to induce a stronger neutralizing response than postfusion F and may be the preferred vaccine antigen [30, 31].

During the COVID-19 pandemic, mRNA and adenoviral vector vaccines have emerged as promising efficacious and safe alternatives to conventional vaccine approaches. These vaccine platforms allow rapid development and affordable scalability and could be readily adapted to new strains or pathogens [32]. Concerns have been raised that vaccination with adenoviral vectors may induce antivector immunity, which may reduce vaccine efficacy after future readministration of the same vector [33, 34]. Heterologous prime-boost approaches with different adenoviral vector types or different vaccine platforms (eg, adenoviral vector followed by mRNA) might circumvent antivector immunity when booster doses are needed to provide durable protection against a pathogen [34–38]. Further improvements of the adenoviral vector platform and future research will shed light on whether the same adenoviral vectors can be administered effectively for different pathogens during a person's life.

A limitation of the study is the restricted racial diversity of the study population (with almost no Black or Asian infants), which limits the generalizability of our results. Additionally, we did not evaluate the administration of 2 low ChAd155-RSV doses and did not analyze cell-mediated immunity. The study was impacted by its partial overlap with the COVID-19 pandemic, when circulation of RSV and other respiratory viruses was reduced, likely as a result of nonpharmaceutical interventions (eg, lockdowns, physical distancing, and masks) implemented to curb the spread of COVID-19 [3–6]. This was most obvious in the second RSV season in our study, when almost no RSV infections were reported. However, the incidence of RSV infection in the first season was high enough for our analysis of the risk of VAERD to be conclusive.

In conclusion, this study showed that the investigational ChAd155-RSV vaccine administered to 6- to 7-month-old, mostly seronaive infants had a reactogenicity and safety profile that was similar to that of other childhood vaccines, with no evidence of VAERD. ChAd155-RSV elicited a neutralizing response that was higher with the high-dose than the low-dose formulation and increased after a second dose. However, development of ChAd155-RSV has been discontinued because the target efficacy profile was unlikely to be met.

## Supplementary Data

Supplementary materials are available at *The Journal of Infectious Diseases* online. Consisting of data provided by the authors to benefit the reader, the posted materials are not copyedited and are the sole responsibility of the authors, so questions or comments should be addressed to the corresponding author.

## Supplementary Material

jiad271_Supplementary_Data

## Notes

***Author contributions.*** A. G. L., E. L.-M., I. D., J. D.-D., N. V., R. M., S. K. S., V. N., W. P., and X. S.-L. were involved in the conception or the design of the study. A. G. L., B. S. C., B. T., C. e., D. F., E. L.-M., E. C. D., E. K., E. J. A., F. M.-T., F. B.-A., F. D. B., G. C. G., H. S., I. S. C., I. D., J. B., J. D. C., J. D.-D., J. M. L., J. P., J. M. M. A., J. T. R. A., K. L., L. S., M. A. M. G., M. M. M.-P., M. M.-P., M. R., N. V., R. M., S. N. F., T. D. L., T. I., T. P., V. N., W. P., X. S.-L., and X. N. participated in the collection or generation of the data. A. G. L., B. S. C., B. T., E. L.-M., E. C. D., E. K., E. J. A., F. M.-T., F. D. B., G. C. G., H. S., I. S. C., J. D. C., J. D.-D., J. B., J. M. L., J. P., J. M. M. A., J. T. R. A., K. L., L. S., M. M.-P., M. R., N. V., S. N. F., T. D. L., T. I., T. P., V. N., W. P., X. S.-L., and X. N. performed the study. B. S. C., D. F., E. J. A., E. K., F. M.-T., Y. Z., I. D., J. D. C., J. M. L., J. M. M. A., M. R., N. V., S. N. F., S. K. S., T. I., T. P., V. N., W. P., X. S.-L., and X. N. were involved in the analyses or interpretation of the data.

***Acknowledgments.*** The authors are grateful to the participants and their parents, the study team and site personnel, and Dr Ilkka Seppa for their contributions to the study. They also thank Akkodis Belgium (c/o GSK) for medical writing and design support (by Natalie Denef) and manuscript coordination.

***Trademark statement.*** Bexsero, Synflorix, and Menveo are trademarks owned by or licensed to GSK. Nimenrix is a trademark owned by or licensed to Pfizer.

***Data availability.*** Study data and documents can be requested for further research from www.clinicalstudydatarequest.com.

***Financial support.*** This work was supported by GlaxoSmithKline Biologicals SA.
