# Supplementary material for: Safety and Immunogenicity of a ChAd155-Vectored Respiratory Syncytial Virus Vaccine in Infants 6–7 Months of age: A Phase 1/2 Randomized Trial
Source: J Infect Dis. 2023 Jul 21;229(1):95–107. doi: 10.1093/infdis/jiad271 (PMC10786261; doi:10.1093/infdis/jiad271)
Supplement: jiad271_Supplementary_Data [file jiad271_supplementary_data.docx]

**Supplementary material**

**Supplementary methods**

***Inclusion criteria***

All infants had to satisfy all the following criteria at study entry:

- Infants’ parents/legally authorized representatives (LARs) who, in the opinion of the investigator, could and would comply with the requirements of the protocol (e.g., completion of the diary cards, return for follow-up visits).
- Written informed consent obtained from the infants’ parents/LARs prior to performing any study-specific procedure.
- A boy or girl between and including 6 and 7 months of age (from the day the infant becomes 6 months of age until the day before the infant becomes 8 months of age) at the time of the first vaccination.
- Healthy infants as established by medical history and clinical examination before entering the study.
- Born full-term (i.e., after a gestation period of 37 to less than 42 completed weeks) with a minimum birth weight of 2.5 kg.
- Infants’ parents/LARs needed to have access to a consistent means of telephone contact (e.g., land line or mobile) or computer.

***Exclusion criteria***

The following criteria had to be checked at the time of study entry. If any exclusion criterion applied, the infant was not included in the study:

- Child in care. This referred to a child who had been placed under the control or protection of an agency, organization, institution, or entity by the courts, the government, or a government body, acting in accordance with powers conferred on them by law or regulation. The definition of a child in care could include a child cared for by foster parents or living in a care home or institution, provided that the arrangement fell within the definition above. The definition of a child in care did not include a child who was adopted or had an appointed legal guardian.
- Use of any investigational or non-registered product (drug or vaccine) other than the study vaccine during the period starting 30 days before the first dose of study vaccine (day -29 to day 1) or planned use during the study period.
- Chronic administration (defined as more than 14 days in total) of immunosuppressants or other immune-modifying drugs during the period starting 6 months before the first vaccination. For corticosteroids, this meant prednisone ≥0.5 mg/kg/day or equivalent. Topical steroids were allowed.
- Administration of long-acting immune-modifying drugs (e.g., infliximab) or planned administration at any time during the study period.
- Administration of immunoglobulins and/or any blood products during the period starting 3 months before the first dose of study vaccine or planned administration during the study period.
- (Planned) administration of a vaccine not foreseen by the study protocol in the period starting 30 days before the first dose and ending 30 days after the last dose of vaccine administration, with the exception of scheduled routine pediatric vaccines. Scheduled routine pediatric vaccines could be administered ≥7 days before a dose of study vaccine or ≥7 days following a dose of study vaccine, with the exception of live viral vaccines, which could be administered ≥14 days before a dose or ≥7 days after a dose.
- Acute or chronic, clinically significant pulmonary, cardiovascular, hepatic, or renal functional abnormality, as determined by physical examination or laboratory screening tests.
- A history of or ongoing confirmed respiratory syncytial virus (RSV) disease or highly compatible clinical picture (e.g., bronchiolitis).
- Serious chronic illness.
- Major congenital defects.
- History of any neurological disorders or seizures.
- History of or current autoimmune disease.
- History of recurrent wheezing (defined as ≥2 episodes of wheezing in the infant´s lifetime). Wheezing should have been verified on auscultation by a doctor.
- History of chronic cough (duration of 4 weeks or more).
- Previous hospitalization for lower respiratory illnesses.
- Previous, current, or planned administration of palivizumab.
- Neurological complications following any prior vaccination.
- Born to a mother known or suspected to be positive for human immunodeficiency virus (no laboratory testing required).
- Any confirmed or suspected immunosuppressive or immunodeficient condition, based on medical history and physical examination (no laboratory testing required).
- Family history of congenital or hereditary immunodeficiency.
- Previous vaccination with a recombinant simian or human adenoviral vaccine.
- History of any reaction or hypersensitivity to any component of the vaccines (investigational or control) or placebo used in this study or any contraindication to them.
- Hypersensitivity to latex.
- Current severe eczema.
- Acute disease and/or fever at the time of enrollment.
  - Fever was defined as a temperature ≥38.0°C/100.4°F. The preferred route for measuring temperature in this study was the rectum for infants <12 months of age.
  - Clinically significant upper respiratory tract infection (RTI).
  - Infants with a minor illness (such as mild diarrhea) without fever could be enrolled at the discretion of the investigator.
- Any clinically significant grade 1 or any grade ≥2 hematological or biochemical laboratory abnormality detected at the last screening blood sampling.
  - For grade 1 laboratory abnormalities, the investigators had to use their clinical judgment to decide which were clinically relevant. Infants with hematological/biochemical values out of the normal range that were expected to be temporary, could be re-screened at a later date.
- Any medical condition that in the judgment of the investigator would make intramuscular injection unsafe.
- Any other conditions that the investigator judged could interfere with study procedures (e.g., drawing blood) or findings (e.g., immune response).
- Any conditions that could constitute a risk for the infants while participating in this study.
- Weight below the fifth percentile according to the World Health Organization weight-for-age tables (<https://www.who.int/childgrowth/standards/weight_for_age/en/>).
- Participating in another clinical study, at any time during the study period, in which the infant or mother (if breastfeeding) was or would be exposed to an investigational or a non-investigational vaccine/product (pharmaceutical product or device).
- Planned move to a location that would prohibit participating in the study until study end.
- For Thailand only, infants who had received the pneumococcal non-typeable *Haemophilus influenzae* protein D conjugate vaccine before enrollment.

***Randomization***

Infants were randomized using a centralized randomization system on internet (SBIR) before first vaccination and after assessment of eligibility. Countries were grouped into 5 levels according to the choice of comparator vaccine or placebo. The randomization algorithm used a minimization procedure accounting for country as a minimization factor and the grouping comparator/placebo as a stratification factor to attempt to maintain a 1:1:1 ratio between the 3 groups (RSV_1D, RSV_2D, comparator) within each level.

***Blinding***

Data were collected in an observer-blinded manner (meaning that the participants’ parents/LARs and those responsible for the evaluation of study endpoints were unaware of which vaccine was administered). Therefore, vaccine preparation and administration were done by authorized medical personnel who did not participate in any of the clinical evaluation assays.

A statistical analysis was performed when all data up to day 61 were collected. As this analysis could lead to the unblinding of some participants, the study could not be considered as observer-blinded after this point and was conducted in a single-blinded manner. The participants’ parents/LARs remained blinded up to the last study visit and the investigators had no access to the treatment allocation up to the last study visit, except in case of emergency unblinding.

The laboratory was blinded to the intervention, and codes were used to link the participant and study (without any link to the intervention attributed to the participant) to each sample.

***Determination of RSV transmission season***

RSV seasons were determined for each country based on local epidemiological data (from before the COVID-19 pandemic) and were defined as the period of the year when 70% of the RSV cases had occurred in previous years. For the calculation, data from up to 10 previous years were applied when available. If the data allowed calculation of the actual date of the beginning of the season rather than an approximation to the nearest month, this was preferred.

***Vaccine administration***

ChAd155-RSV, placebo, and active comparator vaccines were given as 0.5 mL intramuscular injections in the anterolateral thigh. Placebo was formulation buffer S9b containing Na₂HPO₄ (1.3 mg), KH₂PO₄ (186 μg), NaCl (3.85 mg), KCl (100 μg), and MgCl₂ (50 μg).

***Objectives***

*Primary objective*

To evaluate the safety and reactogenicity of the investigational chimpanzee-derived adenoviral vector RSV vaccine (ChAd155-RSV) when administered intramuscularly as 1 dose (1.5x10^10^ viral particles) or as 2 doses (5x10^10^ viral particles) according to a 0, 1-month schedule, up to 60 days after dose 1 (i.e., day 61) in infants aged 6 and 7 months.

- Occurrence of adverse events (AEs) from first vaccination (day 1) up to day 61.
  - Occurrence of each solicited local and general AE, during a 7-day follow-up period after each vaccination (i.e., the day of vaccination and 6 subsequent days).
  - Occurrence of any unsolicited AE, during a 30-day follow-up period after each vaccination (i.e., the day of vaccination and 29 subsequent days).
  - Occurrence of any serious AE (SAE) from day 1 up to day 61.
  - Occurrence of episodes of spontaneous or excessive bleeding (AE of specific interest) during a 30-day follow-up period after each vaccination.

*Secondary objectives*

To evaluate the occurrence of RSV-RTIs of any severity from visit 1 (day 1, after dose 1) up to the end of the first RSV transmission season, in infants aged 6 and 7 months.

- Occurrence of RSV-RTIs, RSV lower respiratory tract infections (LRTIs), severe RSV-LRTIs, and very severe RSV-LRTIs (according to standardized case definitions) from first vaccination (day 1) up to the end of the first RSV transmission season.

To evaluate the occurrence of RSV-RTIs from visit 1 (day 1, after dose 1) up to the end of the second RSV transmission season, in infants aged 6 and 7 months.

- Occurrence of RSV-RTIs, RSV-LRTIs, severe RSV-LRTIs, and very severe RSV-LRTIs (according to standardized case definitions) from first vaccination (day 1) up to the end of the second RSV transmission season.

To evaluate the occurrence of very severe RSV-LRTIs from visit 1 (day 1, after dose 1) up to the end of the first RSV transmission season in RSV-infected infants aged 6 and 7 months who are considered RSV-seronaïve at screening.

- Occurrence of very severe RSV-LRTIs (according to standardized case definitions) among RSV-infected infants who were considered RSV-seronaïve at screening from first vaccination (day 1) up to the end of the first RSV transmission season.

To evaluate the safety of ChAd155-RSV when administered intramuscularly as 1 dose (1.5x10^10^ viral particles) or as 2 doses (5x10^10^ viral particles) according to a 0, 1-month schedule, from study start (day 1) up to the end of the second RSV transmission season in infants aged 6 and 7 months.

- Occurrence of SAEs from first vaccination (day 1) up to the end of the second RSV transmission season.
- Occurrence of RSV-LRTIs (AE of specific interest) from first vaccination (day 1) up to the end of the first RSV transmission season, and up to the end of the second RSV transmission season.

To evaluate the humoral immunogenicity induced by ChAd155-RSV when administered intramuscularly as 1 dose (1.5x10^10^ viral particles) or as 2 doses (5x10^10^ viral particles) according to a 0, 1-month schedule, from study start (day 1) up to the end of the first RSV transmission season, in infants aged 6 and 7 months.

- Humoral response to ChAd155-RSV, pre-vaccination (screening), post-dose 1 (day 31) and post-dose 2 (day 61 and at the end of the first RSV transmission season):
  - Neutralizing titers against RSV-A.
  - RSV F-binding antibody concentrations.

***Safety assessments***

*Adverse events of specific interest*

Spontaneous or excessive bleeding was monitored as an AE of specific interest because in a previous phase 1 study on another investigational adenoviral vector RSV vaccine, a mild, transient decrease in hemoglobin was observed after vaccination [1]. Additionally, a toxicology study with ChAd155-RSV in rabbits showed a transient, non-clinically significant decline in platelets. Parents/LARs had to contact the study staff if their child presented symptoms of spontaneous bleeding or easy bruising or developed a rash within 30 days post-vaccination.

Because of the risk of vaccine-associated enhanced respiratory disease (VAERD) with an investigational formalin-inactivated RSV vaccine [2-5], RSV-LRTIs occurring throughout the study were considered as AEs of specific interest.

*Intensity grading of adverse events*

| **Adverse event** | **Intensity grade** | **Parameter** |
| --- | --- | --- |
| Pain at injection site | 0 | None |
|  | 1 | Mild: minor reaction to touch |
|  | 2 | Moderate: cries/protests on touch |
|  | 3 | Severe: cries when limb is moved/limb is spontaneously painful |
| Redness/swelling at injection site | 0 | None |
|  | 1 | Mild: greatest surface diameter <5 mm |
|  | 2 | Moderate: greatest surface diameter 5–20 mm |
|  | 3 | Severe: greatest surface diameter >20 mm |
| Irritability | 0 | Behavior as usual |
|  | 1 | Mild: crying more than usual/no effect on normal activity |
|  | 2 | Moderate: crying more than usual/interferes with normal activity |
|  | 3 | Severe: crying that cannot be comforted/prevents normal activity |
| Drowsiness | 0 | Behavior as usual |
|  | 1 | Mild: drowsiness easily tolerated |
|  | 2 | Moderate: drowsiness that interferes with normal activity |
|  | 3 | Severe: drowsiness that prevents normal activity |
| Loss of appetite | 0 | Appetite as usual |
|  | 1 | Mild: eating less than usual/no effect on normal activity |
|  | 2 | Moderate: eating less than usual/interferes with normal activity |
|  | 3 | Severe: not eating at all |
| Fever^a^ | 0 | None: temperature <38.0°C/100.4°F |
|  | 1 | Mild: temperature ≥38.0°C/100.4°F to ≤39.0°C/102.2°F |
|  | 2 | Moderate: temperature >39.0°C/102.2°F to ≤40.0°C/104.0°F |
|  | 3 | Severe: temperature >40.0°C/104.0°F |
| Unsolicited adverse event | 1 | Easily tolerated, causing minimal discomfort, and not interfering with everyday activities |
|  | 2 | Sufficiently discomforting to interfere with normal everyday activities |
|  | 3 | Prevents normal, everyday activities, e.g., prevents attendance at school/kindergarten/daycare center and causes the parent(s)/LAR(s) to seek medical advice |

^a^Temperature preferably measured axillary for participants ≥1 year of age and rectally for participants <12 months of age.

***Laboratory assays***

*Quantitative reverse transcription-polymerase chain reaction (RT-PCR)*

RNA extracted from nasal swabs was tested for RSV in a duplex PCR format using specific amplification primers and fluorescent probes designed in the RSV *N* gene, which encodes the RSV nucleocapsid protein. The process involved nucleic acid extraction, conversion of RNA to complementary DNA by reverse transcription, and detection by real-time PCR using a calibration curve (absolute quantitation). The RSV viral load was reported as copies of RSV RNA per mL of sample. Samples were positive for RSV if their viral load was 304 copies/mL for RSV-A or 475 copies/mL for RSV-B. The assay was performed at GSK Clinical Laboratory Sciences in Rixensart or Wavre, Belgium.

***Determination of sample size***

The target sample size of 150 enrolled infants (50 in each group) was based on the minimum number of infants needed to allow detection of a VAERD signal with a magnitude similar to that of the historic formalin-inactivated-RSV vaccine trials [4]. We anticipated that at least 50% of infants would be RSV-seronaïve at screening [6], and we assumed a conservative RSV infection rate of 20% in the first season [7]. Therefore, with 50 infants in each ChAd155-RSV group, we expected to observe at least 5 infected infants who were RSV-seronaïve at screening in each ChAd155-RSV group.

With a 1-sided type I error of 0.05 and the assumption of a 10% rate of infection progressing to very severe RSV-LRTI (which is a conservative assumption based on the rate in the natural history of disease), 5 infections could provide at least 90% statistical power to demonstrate that the progression rate from infection to very severe RSV-LRTI was <80%. This is less extreme than that observed in the historic formalin-inactivated RSV vaccine trial, in which 80% of RSV-RTI cases progressed to hospitalization [4].

If a maximum of 1 out of 5 infections in infants receiving ChAd155-RSV progressed to very severe RSV-LRTI, then it could be concluded with 95% confidence that the expected progression rate of infection was less than the effect reported in previous formalin-inactivated RSV trials; if a minimum of 3 out of 5 infections progressed to very severe RSV-LRTI then it could be concluded with 95% confidence that the expected progression rate of infection was more than the 10% observed in the natural history of infection. This power analysis was based on the procedure of inequality of 1 proportion in PASS 12 software.

With 50 infants in each ChAd155-RSV group, the probability of observing at least 1 SAE would be approximately 92% if the true SAE incidence rate was 5%. To estimate the proportion of infants with AEs, the maximum width of the exact 95% confidence interval would be under 30%, and it could be as large as 35% if the attrition rate was 30%.

**Supplementary results**

***Co-infections***

Co-infections were infrequent. Among participants in the exposed set with an RSV-RTI, 0/23 in the RSV_1D group, 1/18 in the RSV_2D group, and 2/30 in the comparator group had a co-infection (detected with the multiplex PCR respiratory viral panel). One of these episodes (in the comparator group) was a severe RSV-LRTI.

**Supplementary tables**

**Supplementary Table 1. Number (percentage) of participants by country (total enrolled set)**

| **Country** | **RSV_1D**  **N=65** | **RSV_2D**  **N=71** | **Comparator**  **N=65** | **Total**  **N=201** |
| --- | --- | --- | --- | --- |
| Brazil | 5 (7.7) | 6 (8.5) | 5 (7.7) | 16 (8.0) |
| Canada | 3 (4.6) | 6 (8.5) | 3 (4.6) | 12 (6.0) |
| Colombia | 3 (4.6) | 3 (4.2) | 3 (4.6) | 9 (4.5) |
| Finland | 4 (6.2) | 4 (5.6) | 3 (4.6) | 11 (5.5) |
| Italy | 0 (0.0) | 1 (1.4) | 1 (1.5) | 2 (1.0) |
| Mexico | 2 (3.1) | 1 (1.4) | 2 (3.1) | 5 (2.5) |
| Panama | 18 (27.7) | 19 (26.8) | 19 (29.2) | 56 (27.9) |
| Poland | 7 (10.8) | 7 (9.9) | 6 (9.2) | 20 (10.0) |
| Spain | 15 (23.1) | 15 (21.1) | 15 (23.1) | 45 (22.4) |
| Thailand | 1 (1.5) | 1 (1.4) | 1 (1.5) | 3 (1.5) |
| Turkey | 6 (9.2) | 6 (8.5) | 5 (7.7) | 17 (8.5) |
| United Kingdom | 0 (0.0) | 1 (1.4) | 1 (1.5) | 2 (1.0) |
| United States | 1 (1.5) | 1 (1.4) | 1 (1.5) | 3 (1.5) |

RSV, respiratory syncytial virus; RSV_1D, group receiving 1 low ChAd155-RSV dose as dose 1 and placebo as dose 2; RSV_2D, group receiving 2 high ChAd155-RSV doses as dose 1 and 2; comparator, group receiving either placebo as dose 1 and 2, or active comparator vaccine as dose 1 or 2 and placebo as the other dose (pooled); N, total number of enrolled participants.

**Supplementary** **Table 2. Case definitions based on those proposed by the World Health Organization [8]**

| **Case** | **At sea level up to 2500 m elevation** | **Above 2500 m elevation** |
| --- | --- | --- |
| **RSV-RTI** | Rhinorrhea or nasal congestion or cough  and confirmed RSV infection^a^ | Same |
| **RSV-LRTI** | History of cough or difficulty breathing^b^  and SpO_2_ <95%^c^ or RR increase^d^  and confirmed RSV infection^a^ | Same but with SpO_2_ <92%^c^ |
| **Severe RSV-LRTI** | Meeting RSV-LRTI case definition  and SpO_2_ <93%^c^ or lower chest wall indrawing | Same but with SpO_2_ <90%^c^ |
| **Very severe RSV-LRTI** | Meeting RSV-LRTI case definition  and SpO_2_ <90%^c^ or inability to feed or failure to respond/unconscious | Same but with SpO_2_ <88%^c^ |
| **RSV hospitalization** | Confirmed RSV infection^e^  and hospitalized for acute medical condition^f^ | Same |
| **All-cause LRTI** | History of cough or difficulty breathing^b^  and SpO_2_ <95%^c^ or RR increase^d^ | Same but with SpO_2_ <92%^c^ |

RSV, respiratory syncytial virus; (L)RTI, (lower) respiratory tract infection; SpO_2_, blood oxygen saturation; RR, respiratory rate.

^a^Confirmed on nasal swab positive for RSV-A or RSV-B by quantitative reverse transcription-polymerase chain reaction.

^b^Based on history reported by parents.

^c^The lowest value during the illness was used.

^d^RR increase defined as ≥50/minute (2–11 months of age) or ≥40/minute (≥12 months of age). The highest value during the illness was used.

^e^RSV sampling and testing based on medical judgment of medical practitioner or driven by algorithm.

^f^Hospitalization defined as a medical decision that the infant required admission for observation or treatment.

**Supplementary** **Table 3. Solicited local and systemic adverse events within 7 days post-vaccination (exposed set)**

| **Adverse event** | **RSV_1D**  **N=65** | | **RSV_2D**  **N=71** | | **Active comparator**  **N=42** | | **Placebo**  **N=22** | |
| --- | --- | --- | --- | --- | --- | --- | --- | --- |
|  | **n** | **% (95% CI)** | **n** | **% (95% CI)** | **n** | **% (95% CI)** | **n** | **% (95% CI)** |
| **Local adverse events** |  |  |  |  |  |  |  |  |
| **Pain** |  |  |  |  |  |  |  |  |
| **After dose 1** |  |  |  |  |  |  |  |  |
| Any | 11 | 16.9 (8.8–28.3) | 10 | 14.1 (7.0–24.4) | 17 | 40.5 (25.6–56.7) | 1 | 4.5 (0.1–22.8) |
| Grade 2 | 4 | 6.2 (1.7–15.0) | 1 | 1.4 (0.0–7.6) | 5 | 11.9 (4.0–25.6) | 1 | 4.5 (0.1–22.8) |
| Grade 3 | 0 | 0.0 (0.0–5.5) | 1 | 1.4 (0.0–7.6) | 1 | 2.4 (0.1–12.6) | 0 | 0.0 (0.0–15.4) |
| **After dose 2** |  |  |  |  |  |  |  |  |
| Any | 5 | 7.9 (2.6–17.6) | 9 | 12.7 (6.0–22.7) | 6 | 14.6 (5.6–29.2) | 0 | 0.0 (0.0–16.8) |
| Grade 2 | 2 | 3.2 (0.4–11.0) | 1 | 1.4 (0.0–7.6) | 2 | 4.9 (0.6–16.5) | 0 | 0.0 (0.0–16.8) |
| Grade 3 | 0 | 0.0 (0.0–5.7) | 1 | 1.4 (0.0–7.6) | 0 | 0.0 (0.0–8.6) | 0 | 0.0 (0.0–16.8) |
| **After dose 1 or dose 2** |  |  |  |  |  |  |  |  |
| Any | 13 | 20.0 (11.1–31.8) | 12 | 16.9 (9.0–27.7) | 18 | 42.9 (27.7–59.0) | 1 | 4.5 (0.1–22.8) |
| Grade 2 | 5 | 7.7 (2.5–17.0) | 2 | 2.8 (0.3–9.8) | 5 | 11.9 (4.0–25.6) | 1 | 4.5 (0.1–22.8) |
| Grade 3 | 0 | 0.0 (0.0–5.5) | 1 | 1.4 (0.0–7.6) | 1 | 2.4 (0.1–12.6) | 0 | 0.0 (0.0–15.4) |
| **Erythema** |  |  |  |  |  |  |  |  |
| **After dose 1** |  |  |  |  |  |  |  |  |
| Any | 5 | 7.7 (2.5–17.0) | 6 | 8.5 (3.2–17.5) | 22 | 52.4 (36.4–68.0) | 0 | 0.0 (0.0–15.4) |
| Grade 2 | 0 | 0.0 (0.0–5.5) | 2 | 2.8 (0.3–9.8) | 5 | 11.9 (4.0–25.6) | 0 | 0.0 (0.0–15.4) |
| Grade 3 | 0 | 0.0 (0.0–5.5) | 0 | 0.0 (0.0–5.1) | 1 | 2.4 (0.1–12.6) | 0 | 0.0 (0.0–15.4) |
| **After dose 2** |  |  |  |  |  |  |  |  |
| Any | 8 | 12.7 (5.6–23.5) | 7 | 9.9 (4.1–19.3) | 11 | 26.8 (14.2–42.9) | 0 | 0.0 (0.0–16.8) |
| Grade 2 | 0 | 0.0 (0.0–5.7) | 3 | 4.2 (0.9–11.9) | 1 | 2.4 (0.1–12.9) | 0 | 0.0 (0.0–16.8) |
| Grade 3 | 1 | 1.6 (0.0–8.5) | 1 | 1.4 (0.0–7.6) | 0 | 0.0 (0.0–8.6) | 0 | 0.0 (0.0–16.8) |
| **After dose 1 or dose 2** |  |  |  |  |  |  |  |  |
| Any | 9 | 13.8 (6.5–24.7) | 11 | 15.5 (8.0–26.0) | 26 | 61.9 (45.6–76.4) | 0 | 0.0 (0.0–15.4) |
| Grade 2 | 0 | 0.0 (0.0–5.5) | 5 | 7.0 (2.3–15.7) | 6 | 14.3 (5.4–28.5) | 0 | 0.0 (0.0–15.4) |
| Grade 3 | 1 | 1.5 (0.0–8.3) | 1 | 1.4 (0.0–7.6) | 1 | 2.4 (0.1–12.6) | 0 | 0.0 (0.0–15.4) |
| **Swelling** |  |  |  |  |  |  |  |  |
| **After dose 1** |  |  |  |  |  |  |  |  |
| Any | 2 | 3.1 (0.4–10.7) | 3 | 4.2 (0.9–11.9) | 11 | 26.2 (13.9–42.0) | 2 | 9.1 (1.1–29.2) |
| Grade 2 | 0 | 0.0 (0.0–5.5) | 1 | 1.4 (0.0–7.6) | 2 | 4.8 (0.6–16.2) | 1 | 4.5 (0.1–22.8) |
| Grade 3 | 0 | 0.0 (0.0–5.5) | 0 | 0.0 (0.0–5.1) | 2 | 4.8 (0.6–16.2) | 0 | 0.0 (0.0–15.4) |
| **After dose 2** |  |  |  |  |  |  |  |  |
| Any | 1 | 1.6 (0.0–8.5) | 3 | 4.2 (0.9–11.9) | 6 | 14.6 (5.6–29.2) | 0 | 0.0 (0.0–16.8) |
| Grade 2 | 1 | 1.6 (0.0–8.5) | 2 | 2.8 (0.3–9.8) | 0 | 0.0 (0.0–8.6) | 0 | 0.0 (0.0 16.8) |
| Grade 3 | 0 | 0.0 (0.0–5.7) | 0 | 0.0 (0.0–5.1) | 0 | 0.0 (0.0–8.6) | 0 | 0.0 (0.0–16.8) |
| **After dose 1 or dose 2** |  |  |  |  |  |  |  |  |
| Any | 3 | 4.6 (1.0–12.9) | 4 | 5.6 (1.6–13.8) | 16 | 38.1 (23.6–54.4) | 2 | 9.1 (1.1–29.2) |
| Grade 2 | 1 | 1.5 (0.0–8.3) | 3 | 4.2 (0.9–11.9) | 2 | 4.8 (0.6–16.2) | 1 | 4.5 (0.1–22.8) |
| Grade 3 | 0 | 0.0 (0.0–5.5) | 0 | 0.0 (0.0–5.1) | 2 | 4.8 (0.6–16.2) | 0 | 0.0 (0.0–15.4) |
| **Systemic adverse events** |  |  |  |  |  |  |  |  |
| **Loss of appetite** |  |  |  |  |  |  |  |  |
| **After dose 1** |  |  |  |  |  |  |  |  |
| Any | 12 | 18.5 (9.9–30.0) | 17 | 23.9 (14.6–35.5) | 14 | 33.3 (19.6–49.5) | 8 | 36.4 (17.2–59.3) |
| Grade 2 | 3 | 4.6 (1.0–12.9) | 3 | 4.2 (0.9–11.9) | 2 | 4.8 (0.6–16.2) | 4 | 18.2 (5.2–40.3) |
| Grade 3 | 1 | 1.5 (0.0–8.3) | 1 | 1.4 (0.0–7.6) | 1 | 2.4 (0.1–12.6) | 1 | 4.5 (0.1–22.8) |
| **After dose 2** |  |  |  |  |  |  |  |  |
| Any | 7 | 11.1 (4.6–21.6) | 22 | 31.0 (20.5–43.1) | 8 | 19.5 (8.8–34.9) | 3 | 15.0 (3.2–37.9) |
| Grade 2 | 2 | 3.2 (0.4–11.0) | 4 | 5.6 (1.6–13.8) | 1 | 2.4 (0.1–12.9) | 0 | 0.0 (0.0–16.8) |
| Grade 3 | 0 | 0.0 (0.0–5.7) | 0 | 0.0 (0.0–5.1) | 2 | 4.9 (0.6–16.5) | 0 | 0.0 (0.0–16.8) |
| **After dose 1 or dose 2** |  |  |  |  |  |  |  |  |
| Any | 17 | 26.2 (16.0–38.5) | 32 | 45.1 (33.2–57.3) | 17 | 40.5 (25.6–56.7) | 8 | 36.4 (17.2–59.3) |
| Grade 2 | 4 | 6.2 (1.7–15.0) | 6 | 8.5 (3.2–17.5) | 2 | 4.8 (0.6–16.2) | 4 | 18.2 (5.2–40.3) |
| Grade 3 | 1 | 1.5 (0.0–8.3) | 1 | 1.4 (0.0–7.6) | 3 | 7.1 (1.5–19.5) | 1 | 4.5 (0.1–22.8) |
| **Irritability** |  |  |  |  |  |  |  |  |
| **After dose 1** |  |  |  |  |  |  |  |  |
| Any | 25 | 38.5 (26.7–51.4) | 31 | 43.7 (31.9–56.0) | 25 | 59.5 (43.3–74.4) | 9 | 40.9 (20.7–63.6) |
| Grade 2 | 3 | 4.6 (1.0–12.9) | 8 | 11.3 (5.0–21.0) | 10 | 23.8 (12.1–39.5) | 4 | 18.2 (5.2–40.3) |
| Grade 3 | 3 | 4.6 (1.0–12.9) | 1 | 1.4 (0.0–7.6) | 1 | 2.4 (0.1–12.6) | 2 | 9.1 (1.1–29.2) |
| **After dose 2** |  |  |  |  |  |  |  |  |
| Any | 18 | 28.6 (17.9–41.3) | 33 | 46.5 (34.5–58.7) | 15 | 36.6 (22.1–53.1) | 3 | 15.0 (3.2–37.9) |
| Grade 2 | 11 | 17.5 (9.1–29.1) | 13 | 18.3 (10.1–29.3) | 3 | 7.3 (1.5–19.9) | 2 | 10.0 (1.2–31.7) |
| Grade 3 | 1 | 1.6 (0.0–8.5) | 2 | 2.8 (0.3–9.8) | 4 | 9.8 (2.7–23.1) | 0 | 0.0 (0.0–16.8) |
| **After dose 1 or dose 2** |  |  |  |  |  |  |  |  |
| Any | 34 | 52.3 (39.5–64.9) | 41 | 57.7 (45.4–69.4) | 27 | 64.3 (48.0–78.4) | 9 | 40.9 (20.7–63.6) |
| Grade 2 | 12 | 18.5 (9.9–30.0) | 18 | 25.4 (15.8–37.1) | 9 | 21.4 (10.3–36.8) | 6 | 27.3 (10.7–50.2) |
| Grade 3 | 4 | 6.2 (1.7–15.0) | 3 | 4.2 (0.9–11.9) | 4 | 9.5 (2.7–22.6) | 2 | 9.1 (1.1–29.2) |
| **Drowsiness** |  |  |  |  |  |  |  |  |
| **After dose 1** |  |  |  |  |  |  |  |  |
| Any | 12 | 18.5 (9.9–30.0) | 19 | 26.8 (16.9–38.6) | 14 | 33.3 (19.6–49.5) | 7 | 31.8 (13.9–54.9) |
| Grade 2 | 1 | 1.5 (0.0–8.3) | 4 | 5.6 (1.6–13.8) | 3 | 7.1 (1.5–19.5) | 3 | 13.6 (2.9–34.9) |
| Grade 3 | 1 | 1.5 (0.0–8.3) | 1 | 1.4 (0.0–7.6) | 0 | 0.0 (0.0–8.4) | 0 | 0.0 (0.0–15.4) |
| **After dose 2** |  |  |  |  |  |  |  |  |
| Any | 10 | 15.9 (7.9–27.3) | 18 | 25.4 (15.8–37.1) | 9 | 22.0 (10.6–37.6) | 3 | 15.0 (3.2–37.9) |
| Grade 2 | 2 | 3.2 (0.4–11.0) | 4 | 5.6 (1.6–13.8) | 0 | 0.0 (0.0–8.6) | 0 | 0.0 (0.0–16.8) |
| Grade 3 | 1 | 1.6 (0.0–8.5) | 3 | 4.2 (0.9–11.9) | 3 | 7.3 (1.5–19.9) | 0 | 0.0 (0.0–16.8) |
| **After dose 1 or dose 2** |  |  |  |  |  |  |  |  |
| Any | 18 | 27.7 (17.3–40.2) | 29 | 40.8 (29.3–53.2) | 18 | 42.9 (27.7–59.0) | 8 | 36.4 (17.2–59.3) |
| Grade 2 | 2 | 3.1 (0.4–10.7) | 7 | 9.9 (4.1–19.3) | 2 | 4.8 (0.6–16.2) | 3 | 13.6 (2.9–34.9) |
| Grade 3 | 2 | 3.1 (0.4–10.7) | 4 | 5.6 (1.6–13.8) | 3 | 7.1 (1.5–19.5) | 0 | 0.0 (0.0–15.4) |
| **Fever** |  |  |  |  |  |  |  |  |
| **After dose 1** |  |  |  |  |  |  |  |  |
| Any | 9 | 13.8 (6.5–24.7) | 24 | 33.8 (23.0–46.0) | 13 | 31.0 (17.6–47.1) | 5 | 22.7 (7.8–45.4) |
| Grade 2 | 1 | 1.5 (0.0–8.3) | 3 | 4.2 (0.9–11.9) | 1 | 2.4 (0.1–12.6) | 0 | 0.0 (0.0–15.4) |
| Grade 3 | 0 | 0.0 (0.0–5.5) | 0 | 0.0 (0.0–5.1) | 1 | 2.4 (0.1–12.6) | 0 | 0.0 (0.0–15.4) |
| **After dose 2** |  |  |  |  |  |  |  |  |
| Any | 6 | 9.5 (3.6–19.6) | 28 | 39.4 (28.0–51.7) | 4 | 9.8 (2.7–23.1) | 0 | 0.0 (0.0–16.8) |
| Grade 2 | 1 | 1.6 (0.0–8.5) | 6 | 8.5 (3.2–17.5) | 3 | 7.3 (1.5–19.9) | 0 | 0.0 (0.0–16.8) |
| Grade 3 | 0 | 0.0 (0.0–5.7) | 1 | 1.4 (0.0–7.6) | 0 | 0.0 (0.0–8.6) | 0 | 0.0 (0.0–16.8) |
| **After dose 1 or dose 2** |  |  |  |  |  |  |  |  |
| Any | 15 | 23.1 (13.5–35.2) | 37 | 52.1 (39.9–64.1) | 16 | 38.1 (23.6–54.4) | 5 | 22.7 (7.8–45.4) |
| Grade 2 | 2 | 3.1 (0.4–10.7) | 8 | 11.3 (5.0–21.0) | 4 | 9.5 (2.7–22.6) | 0 | 0.0 (0.0–15.4) |
| Grade 3 | 0 | 0.0 (0.0–5.5) | 1 | 1.4 (0.0–7.6) | 1 | 2.4 (0.1–12.6) | 0 | 0.0 (0.0–15.4) |

See “Safety assessments” section in this Supplementary material for definitions of grade 2 and grade 3 adverse events.

RSV, respiratory syncytial virus; RSV_1D, group receiving 1 low chimpanzee-derived replication-deficient adenoviral vector RSV vaccine (ChAd155-RSV) dose as dose 1 and placebo as dose 2; RSV_2D, group receiving 2 high ChAd155-RSV doses as dose 1 and 2; active comparator, group receiving active comparator vaccine as dose 1 or 2 and placebo as the other dose; placebo, group receiving placebo as dose 1 and 2; N, total number of participants with available results; the numbers of participants who received dose 2 and had available results were 63 (RSV_1D), 71 (RSV_2D), 41 (active comparator), and 20 (placebo); n/%, number/percentage of participants presenting the adverse event at least once; CI, confidence interval.

**Supplementary** **Table 4. Serious adverse events reported from dose 1 until the end of the second RSV season, by MedDRA primary system organ class and preferred term (exposed set)**

| **Primary system organ class**  Preferred term |  | **RSV_1D**  **N=65** |  | **RSV_2D**  **N=71** |  | **Comparator**  **N=65** |
| --- | --- | --- | --- | --- | --- | --- |
|  | **n** | **% (95% CI)** | **n** | **% (95% CI)** | **n** | **% (95% CI)** |
| **At least one SAE** | 7 | 10.8 (4.4–20.9) | 11 | 15.5 (8.0–26.0) | 3 | 4.6 (1.0–12.9) |
| **Gastrointestinal disorders** | 1 | 1.5 (0.0–8.3) | 0 | 0.0 (0.0–5.1) | 0 | 0.0 (0.0–5.5) |
| Gastrointestinal hemorrhage^a^ | 1 | 1.5 (0.0–8.3) | 0 | 0.0 (0.0–5.1) | 0 | 0.0 (0.0–5.5) |
| **Infections and infestations** | 5 | 7.7 (2.5–17.0) | 9 | 12.7 (6.0–22.7) | 3 | 4.6 (1.0–12.9) |
| Abscess neck | 1 | 1.5 (0.0–8.3) | 0 | 0.0 (0.0–5.1) | 0 | 0.0 (0.0–5.5) |
| Bronchiolitis | 0 | 0.0 (0.0–5.5) | 1 | 1.4 (0.0–7.6) | 0 | 0.0 (0.0–5.5) |
| Gastroenteritis | 2 | 3.1 (0.4–10.7) | 0 | 0.0 (0.0–5.1) | 0 | 0.0 (0.0–5.5) |
| Gastroenteritis viral | 0 | 0.0 (0.0–5.5) | 1 | 1.4 (0.0–7.6) | 0 | 0.0 (0.0–5.5) |
| H1N1 influenza | 1 | 1.5 (0.0–8.3) | 1 | 1.4 (0.0–7.6) | 0 | 0.0 (0.0–5.5) |
| Infectious mononucleosis | 0 | 0.0 (0.0–5.5) | 0 | 0.0 (0.0–5.1) | 1 | 1.5 (0.0–8.3) |
| Lower respiratory tract infection viral | 0 | 0.0 (0.0–5.5) | 1 | 1.4 (0.0–7.6) | 0 | 0.0 (0.0–5.5) |
| Mastoiditis | 0 | 0.0 (0.0–5.5) | 1 | 1.4 (0.0–7.6) | 0 | 0.0 (0.0–5.5) |
| Parvovirus infection | 0 | 0.0 (0.0–5.5) | 1 | 1.4 (0.0–7.6) | 0 | 0.0 (0.0–5.5) |
| Periorbital cellulitis | 0 | 0.0 (0.0–5.5) | 1 | 1.4 (0.0–7.6) | 0 | 0.0 (0.0–5.5) |
| Pneumonia | 1 | 1.5 (0.0–8.3) | 2 | 2.8 (0.3–9.8) | 0 | 0.0 (0.0–5.5) |
| Pneumonia RSV | 1 | 1.5 (0.0–8.3) | 0 | 0.0 (0.0–5.1) | 0 | 0.0 (0.0–5.5) |
| RSV bronchiolitis | 0 | 0.0 (0.0–5.5) | 1 | 1.4 (0.0–7.6) | 1 | 1.5 (0.0–8.3) |
| Urinary tract infection | 0 | 0.0 (0.0–5.5) | 0 | 0.0 (0.0–5.1) | 1 | 1.5 (0.0–8.3) |
| **Injury, poisoning, and procedural complications** | 0 | 0.0 (0.0–5.5) | 1 | 1.4 (0.0–7.6) | 0 | 0.0 (0.0–5.5) |
| Foreign body in respiratory tract | 0 | 0.0 (0.0–5.5) | 1 | 1.4 (0.0–7.6) | 0 | 0.0 (0.0–5.5) |
| **Metabolism and nutrition disorders** | 0 | 0.0 (0.0–5.5) | 1 | 1.4 (0.0–7.6) | 0 | 0.0 (0.0–5.5) |
| Diabetic ketoacidosis^a^ | 0 | 0.0 (0.0–5.5) | 1 | 1.4 (0.0–7.6) | 0 | 0.0 (0.0–5.5) |
| **Psychiatric disorders** | 0 | 0.0 (0.0–5.5) | 1 | 1.4 (0.0–7.6) | 0 | 0.0 (0.0–5.5) |
| Insomnia | 0 | 0.0 (0.0–5.5) | 1 | 1.4 (0.0–7.6) | 0 | 0.0 (0.0–5.5) |
| **Respiratory, thoracic, and mediastinal disorders** | 1 | 1.5 (0.0–8.3) | 0 | 0.0 (0.0–5.1) | 0 | 0.0 (0.0–5.5) |
| Asthma | 1 | 1.5 (0.0–8.3) | 0 | 0.0 (0.0–5.1) | 0 | 0.0 (0.0–5.5) |

RSV, respiratory syncytial virus; MedDRA, Medical Dictionary for Regulatory Activities; RSV_1D, group receiving 1 low chimpanzee-derived replication-deficient adenoviral vector RSV vaccine (ChAd155-RSV) dose as dose 1 and placebo as dose 2; RSV_2D, group receiving 2 high ChAd155-RSV doses as dose 1 and 2; comparator, group receiving either placebo as dose 1 and 2, or active comparator vaccine as dose 1 or 2 and placebo as the other dose (pooled); N, total number of participants in the exposed set; n/%, number/percentage of participants reporting the adverse event at least once; CI, confidence interval; SAE, serious adverse event.

^a^These events were considered by the investigator as possibly related to the ChAd155-RSV vaccine:

*Gastrointestinal hemorrhage*

A case of gastrointestinal hemorrhage occurred in a male participant aged 7 months in the RSV_1D group who presented 16 days after ChAd155-RSV vaccination with bloody stool, anal fissure, and a history of fever and diarrhea. He was found with a low hemoglobin level of 9.9 g/dL, a high platelet count of 436000/µL, a low activated partial thromboplastin time of 27.7 s (normal range: 30.6–39.9), and a normal international normalized ratio. The participant had an elevated white blood cell count and C-reactive protein level. The gastrointestinal bleeding stopped the day after ceftriaxone and metronidazole were started. The blood culture was positive for skin flora only, and the stool culture was negative. The investigator reported the event as possibly related to vaccination because “spontaneous or excessive bleeding” was defined as an adverse event of specific interest in this trial and an infectious etiology of the event was not definitively proven. However, given the probable infectious etiology, the presence of anal fissure, and the increased platelet count, the sponsor considered this event unlikely to be related to vaccination.

*Diabetic ketoacidosis*

Approximately 1 year after the second ChAd155-RSV dose and 55 days after the last dose of 4CMenB, a 20-month-old male participant (who had received 2 doses of ChAd155-RSV, followed by 3 doses of 4CMenB) was hospitalized for new-onset diabetes mellitus and diabetic ketoacidosis. The child also had a rash and fever 1 week before the onset of diabetic ketoacidosis, but no additional tests were performed for the rash and no specific diagnosis was offered. In addition, since enrolling in the study, the child had a mild RSV infection, mastoiditis, and an undiagnosed upper respiratory infection (possibly COVID-19). No known family history or genetic risk factors for diabetes were identified. The child tested positive for diabetes mellitus type 1 autoantibodies (anti-GAD65 antibodies: 2.1 IU/mL, anti-tyrosine phosphatase IA2 antibodies: 149 U/mL). While immune-stimulating events could contribute to the presentation of underlying diabetes, to date large metanalyses have not found evidence to support a link between vaccination and pediatric diabetes mellitus. In addition, the rash and fever 1 week before onset are suggestive of an alternate infectious/viral etiology, which may have triggered an immune/inflammatory response contributing to the presentation of underlying diabetes mellitus. Given the time-to-onset and alternate infectious/inflammatory etiology as an event contributing to the presentation of diabetes mellitus, the sponsor did not consider this event related to the investigational vaccine.

**Supplementary** **Table 5. Serious adverse events with onset within 30 days after first or second vaccination, by MedDRA primary system organ class and preferred term (exposed set)**

| **Primary system organ class**  Preferred term |  | **RSV_1D**  **N=65** |  | **RSV_2D**  **N=71** |  | **Comparator**  **N=65** |
| --- | --- | --- | --- | --- | --- | --- |
|  | **n** | **% (95% CI)** | **n** | **% (95% CI)** | **n** | **% (95% CI)** |
| **At least one SAE** | 3 | 4.6 (1.0–12.9) | 3 | 4.2 (0.9–11.9) | 1 | 1.5 (0.0–8.3) |
| **Gastrointestinal disorders** | 1 | 1.5 (0.0–8.3) | 0 | 0.0 (0.0–5.1) | 0 | 0.0 (0.0–5.5) |
| Gastrointestinal hemorrhage | 1 | 1.5 (0.0–8.3) | 0 | 0.0 (0.0–5.1) | 0 | 0.0 (0.0–5.5) |
| **Infections and infestations** | 2 | 3.1 (0.4–10.7) | 3 | 4.2 (0.9–11.9) | 1 | 1.5 (0.0–8.3) |
| Bronchiolitis | 0 | 0.0 (0.0–5.5) | 1 | 1.4 (0.0–7.6) | 0 | 0.0 (0.0–5.5) |
| Parvovirus infection | 0 | 0.0 (0.0–5.5) | 1 | 1.4 (0.0–7.6) | 0 | 0.0 (0.0–5.5) |
| Pneumonia | 1 | 1.5 (0.0–8.3) | 1 | 1.4 (0.0–7.6) | 0 | 0.0 (0.0–5.5) |
| Pneumonia RSV | 1 | 1.5 (0.0–8.3) | 0 | 0.0 (0.0–5.1) | 0 | 0.0 (0.0–5.5) |
| RSV bronchiolitis | 0 | 0.0 (0.0–5.5) | 0 | 0.0 (0.0–5.1) | 1 | 1.5 (0.0–8.3) |

In the RSV_1D group, gastrointestinal hemorrhage started 16 days after ChAd155-RSV, pneumonia 8 days after ChAd155-RSV, and pneumonia RSV 10 days after placebo. In the RSV_2D group, parvovirus infection started 1 day after the second ChAd155-RSV dose, bronchiolitis 18 days after the second ChAd155-RSV dose, and pneumonia 27 days after the second ChAd155-RSV dose. In the comparator group, RSV bronchiolitis started 29 days after the comparator vaccine dose.

MedDRA, Medical Dictionary for Regulatory Activities; RSV, respiratory syncytial virus; RSV_1D, group receiving 1 low chimpanzee-derived replication-deficient adenoviral vector RSV vaccine (ChAd155-RSV) dose as dose 1 and placebo as dose 2; RSV_2D, group receiving 2 high ChAd155-RSV doses as dose 1 and 2; comparator, group receiving either placebo as dose 1 and 2, or active comparator vaccine as dose 1 or 2 and placebo as the other dose (pooled); N, total number of participants in the exposed set; n/%, number/percentage of participants reporting the adverse event at least once; CI, confidence interval; SAE, serious adverse event.

**Supplementary** **Table 6. RSV surveillance from dose 1 until the end of the second RSV season (exposed set)**

| **Category** | **RSV_1D**  **N=65** | | **RSV_2D**  **N=71** | | **Comparator**  **N=65** | |
| --- | --- | --- | --- | --- | --- | --- |
|  | **n** | **% (95% CI)** | **n** | **% (95% CI)** | **n** | **% (95% CI)** |
| No infection | 38 | 58.5 (45.6–70.6) | 51 | 71.8 (59.9–81.9) | 33 | 50.8 (38.1–63.4) |
| RSV infection (symptomatic or asymptomatic)^a^ | 27 | 41.5 (29.4–54.4) | 20 | 28.2 (18.1–40.1) | 32 | 49.2 (36.6–61.9) |
| RSV-RTI^b^ | 23 | 35.4 (23.9–48.2) | 18 | 25.4 (15.8–37.1) | 30 | 46.2 (33.7–59.0) |
| RSV-LRTI | 3 | 4.6 (1.0–12.9) | 3 | 4.2 (0.9–11.9) | 4 | 6.2 (1.7–15.0) |
| Severe RSV-LRTI | 1 | 1.5 (0.0–8.3) | 1 | 1.4 (0.0–7.6) | 3 | 4.6 (1.0–12.9) |
| Very severe RSV-LRTI | 0 | 0.0 (0.0–5.5) | 0 | 0.0 (0.0–5.1) | 0 | 0.0 (0.0–5.5) |
| All-cause LRTI | 14 | 21.5 (12.3–33.5) | 14 | 19.7 (11.2–30.9) | 11 | 16.9 (8.8–28.3) |
| RSV hospitalization^c^ | 1 | 1.5 (0.0–8.3) | 1 | 1.4 (0.0–7.6) | 1 | 1.5 (0.0–8.3) |
| RSV-LRTI hospitalization^c^ | 1 | 1.5 (0.0–8.3) | 0 | 0.0 (0.0–5.1) | 1 | 1.5 (0.0–8.3) |
| Severe RSV-LRTI hospitalization^c^ | 1 | 1.5 (0.0–8.3) | 0 | 0.0 (0.0–5.1) | 1 | 1.5 (0.0–8.3) |
| Very severe RSV-LRTI hospitalization^c^ | 0 | 0.0 (0.0–5.5) | 0 | 0.0 (0.0–5.1) | 0 | 0.0 (0.0–5.5) |
| All-cause LRTI hospitalization | 2 | 3.1 (0.4–10.7) | 1 | 1.4 (0.0–7.6) | 1 | 1.5 (0.0–8.3) |

RSV, respiratory syncytial virus; RSV_1D, group receiving 1 low chimpanzee-derived replication-deficient adenoviral vector RSV vaccine (ChAd155-RSV) dose as dose 1 and placebo as dose 2; RSV_2D, group receiving 2 high ChAd155-RSV doses as dose 1 and 2; comparator, group receiving either placebo as dose 1 and 2, or active comparator vaccine as dose 1 or 2 and placebo as the other dose (pooled); N, total number of participants in the exposed set; n/%, number/percentage of participants meeting the specified case definition at least once (case definitions were based on those proposed by the World Health Organization, see **Supplementary** **Table 2**); CI, confidence interval; (L)RTI, (lower) respiratory tract infection.

^a^All confirmed RSV infections based on central reverse transcription-polymerase chain reaction testing.

^b^This included both upper and lower respiratory tract infections. Note, all but one RSV-RTI case in the RSV_1D group, all but one in the RSV_2D group, and all RSV-RTI cases in the comparator group occurred after dose 2 (which was expected, given the shorter time interval between the 2 doses [1 month] compared to after dose 2 [almost 2 years] and given the fact that dose 1 was administered before the start of the RSV season).

^c^Confirmed RSV infection and hospitalized for acute medical condition.

**Supplementary** **Table 7. RSV-A neutralizing titers and RSV F-binding IgG concentrations (per-protocol population)**

| **Timepoint** | **RSV_1D** | | **RSV_2D** | | **Comparator** | |
| --- | --- | --- | --- | --- | --- | --- |
| **RSV-A neutralizing titers** |  |  |  |  |  |  |
| **% ≥ assay cut-off** | **N** | **% (95% CI)** | **N** | **% (95% CI)** | **N** | **% (95% CI)** |
| Baseline | 64 | 59.4 (46.4–71.5) | 71 | 73.2 (61.4–83.1) | 61 | 77.0 (64.5–86.8) |
| Day 31 | 63 | 90.5 (80.4–96.4) | 69 | 100 (94.8–100) | 61 | 49.2 (36.1–62.3) |
| Day 61 | 63 | 88.9 (78.4–95.4) | 70 | 100 (94.9–100) | 56 | 33.9 (21.8–47.8) |
| End RSV season 1 | 60 | 76.7 (64.0–86.6) | 70 | 100 (94.9–100) | 61 | 59.0 (45.7–71.4) |
| **GMT** | **N** | **GMT (95% CI), ED60** | **N** | **GMT (95% CI), ED60** | **N** | **GMT (95% CI), ED60** |
| Baseline | 64 | 26.8 (20.7–34.6) | 71 | 29.6 (23.6–37.3) | 61 | 32.2 (24.9–41.6) |
| Day 31 | 63 | 60.2 (44.2–81.9) | 69 | 116.2 (87.6–153.9) | 61 | 18.9 (14.8–24.1) |
| Day 61 | 63 | 54.3 (37.7–78.0) | 70 | 259.4 (211.6–318.1) | 56 | 14.4 (11.8–17.7) |
| End RSV season 1 | 60 | 165.0 (95.0–286.6) | 70 | 223.7 (154.7–323.4) | 61 | 66.3 (40.2–109.5) |
| **GMR** | **N** | **GMR (95% CI)** | **N** | **GMR (95% CI)** | **N** | **GMR (95% CI)** |
| Day 31/baseline | 63 | 2.29 (1.58–3.31) | 69 | 3.95 (2.94–5.32) | 61 | 0.59 (0.49–0.70) |
| Day 61/baseline | 63 | 1.99 (1.28–3.10) | 70 | 8.83 (6.54–11.93) | 56 | 0.44 (0.37–0.52) |
| End RSV season 1/baseline | 60 | 6.28 (3.40–11.60) | 70 | 7.83 (5.19–11.81) | 61 | 2.06 (1.19–3.56) |
| **RSV F-binding IgG (ELISA)** |  |  |  |  |  |  |
| **% ≥ assay cut-off** | **N** | **% (95% CI)** | **N** | **% (95% CI)** | **N** | **% (95% CI)** |
| Baseline | 63 | 95.2 (86.7–99.0) | 71 | 87.3 (77.3–94.0) | 61 | 90.2 (79.8–96.3) |
| Day 31 | 64 | 100 (94.4–100) | 70 | 98.6 (92.3–100) | 60 | 65.0 (51.6–76.9) |
| Day 61 | 61 | 100 (94.1–100) | 70 | 100 (94.9–100) | 55 | 41.8 (28.7–55.9) |
| End RSV season 1 | 60 | 100 (94.0–100) | 69 | 100 (94.8–100) | 60 | 63.3 (49.9–75.4) |
| **GMC** | **N** | **GMC (95% CI), EU/mL** | **N** | **GMC (95% CI), EU/mL** | **N** | **GMC (95% CI), EU/mL** |
| Baseline | 63 | 93.1 (72.7–119.1) | 71 | 81.9 (61.8–108.6) | 61 | 86.0 (65.5–112.7) |
| Day 31 | 64 | 2035.2 (1490.0–2779.9) | 70 | 4550.8 (3354.6–6173.7) | 60 | 46.2 (31.6–67.6) |
| Day 61 | 61 | 1976.5 (1346.2–2901.8) | 70 | 9287.9 (7885.5–10939.7) | 55 | 24.6 (18.3–33.0) |
| End RSV season 1 | 60 | 5108.7 (3096.7–8428.0) | 69 | 4935.5 (3639.8–6692.4) | 60 | 345.1 (165.9–717.7) |
| **GMR** | **N** | **GMR (95% CI)** | **N** | **GMR (95% CI)** | **N** | **GMR (95% CI)** |
| Day 31/baseline | 63 | 22.34 (15.06–33.15) | 70 | 55.53 (38.02–81.12) | 60 | 0.53 (0.41–0.69) |
| Day 61/baseline | 60 | 21.76 (13.62–34.77) | 70 | 113.38 (82.32–156.15) | 55 | 0.27 (0.22–0.35) |
| End RSV season 1/baseline | 59 | 55.58 (29.84–103.52) | 69 | 61.54 (42.00–90.17) | 60 | 4.02 (1.93–8.35) |

RSV, respiratory syncytial virus; RSV-A, RSV subtype A; RSV F, RSV fusion protein; RSV_1D, group receiving 1 low chimpanzee-derived replication-deficient adenoviral vector RSV vaccine (ChAd155-RSV) dose as dose 1 and placebo as dose 2; RSV_2D, group receiving 2 high ChAd155-RSV doses as dose 1 and 2; comparator, group receiving either placebo as dose 1 and 2, or active comparator vaccine as dose 1 or 2 and placebo as the other dose (pooled); N, total number of participants in the per-protocol population with available results at the indicated timepoint (for GMRs, at the indicated post-vaccination timepoint and at baseline); % ≥ assay cut-off, percentage of participants with an RSV-A neutralizing titer ≥18 ED60 (lower limit of quantification) or an RSV F-binding IgG antibody concentration ≥25 EU/mL (limit of detection) measured by enzyme-linked immunosorbent assay (ELISA); CI, confidence interval; GMT, geometric mean titer; ED60, estimated dilution 60; GMR, geometric mean of individual ratios of titers/concentrations at the indicated post-vaccination timepoint over baseline; IgG, immunoglobulin G; GMC, geometric mean concentration; EU, ELISA units.

**Supplementary figures**

**Supplementary Figure 1. Overview of active and passive surveillance and assessment visits**


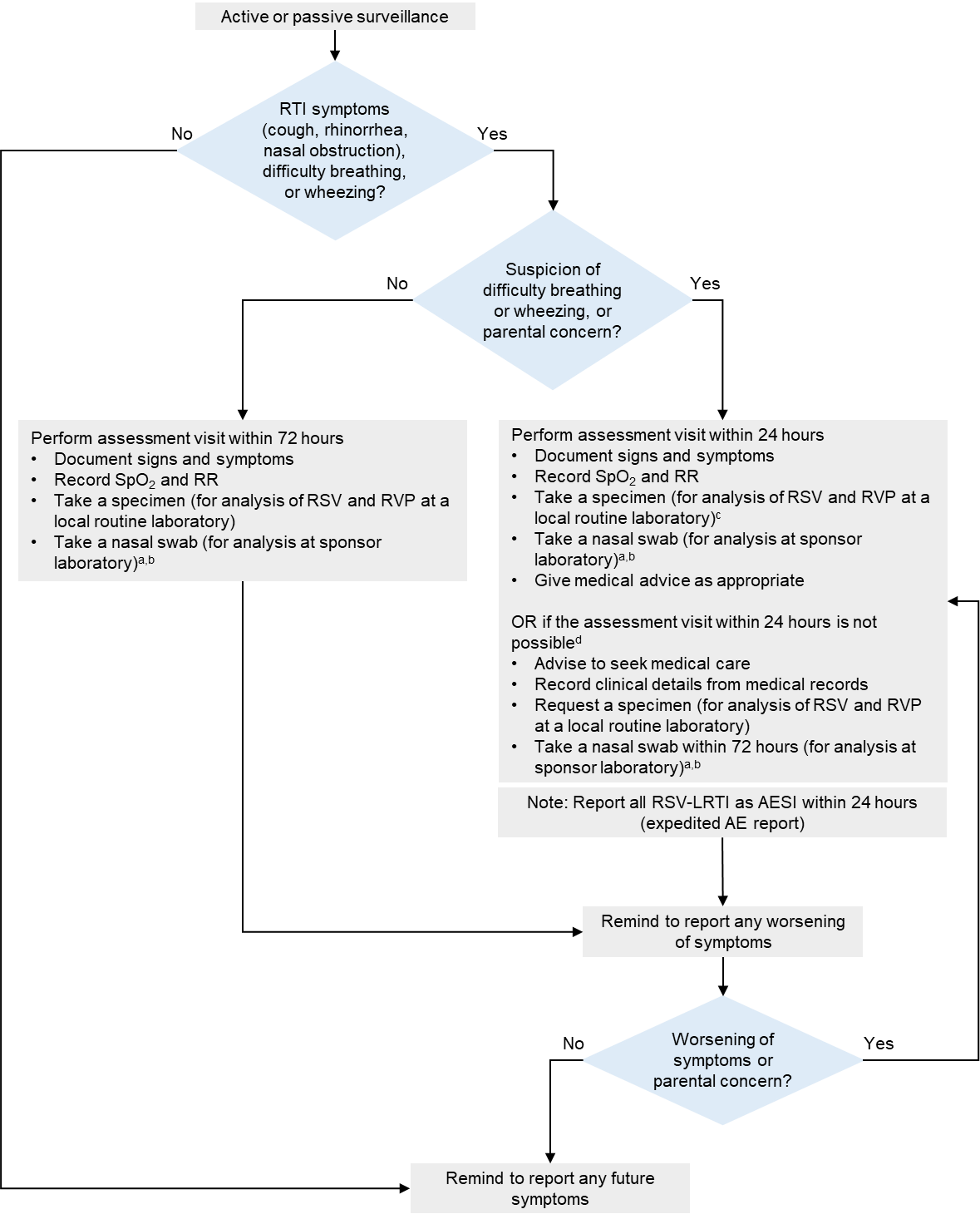


RTI, respiratory tract infection; SpO_2_, oxygen saturation; RR, respiratory rate; RSV, respiratory syncytial virus; RVP, respiratory viral panel; LRTI, lower respiratory tract infection; AESI, adverse event of specific interest; AE, adverse event.

^a^RSV-A/B quantitative reverse transcription-polymerase chain reaction on all specimens.

^b^Respiratory viral panel (multiplex polymerase chain reaction) on all specimens that were RSV-A/B-positive and on all cases of confirmed LRTI according to the case definition.

^c^In case of worsening of symptoms, this sample was optional if the previous specimen was RSV-positive and mandatory if the previous sample was RSV-negative.

^d^For example, if the participant requires urgent medical evaluation and care or has traveled to another location.

**Supplementary Figure 2. Percentage of infants with fever within 7 days after dose 1 or dose 2 (exposed set)**


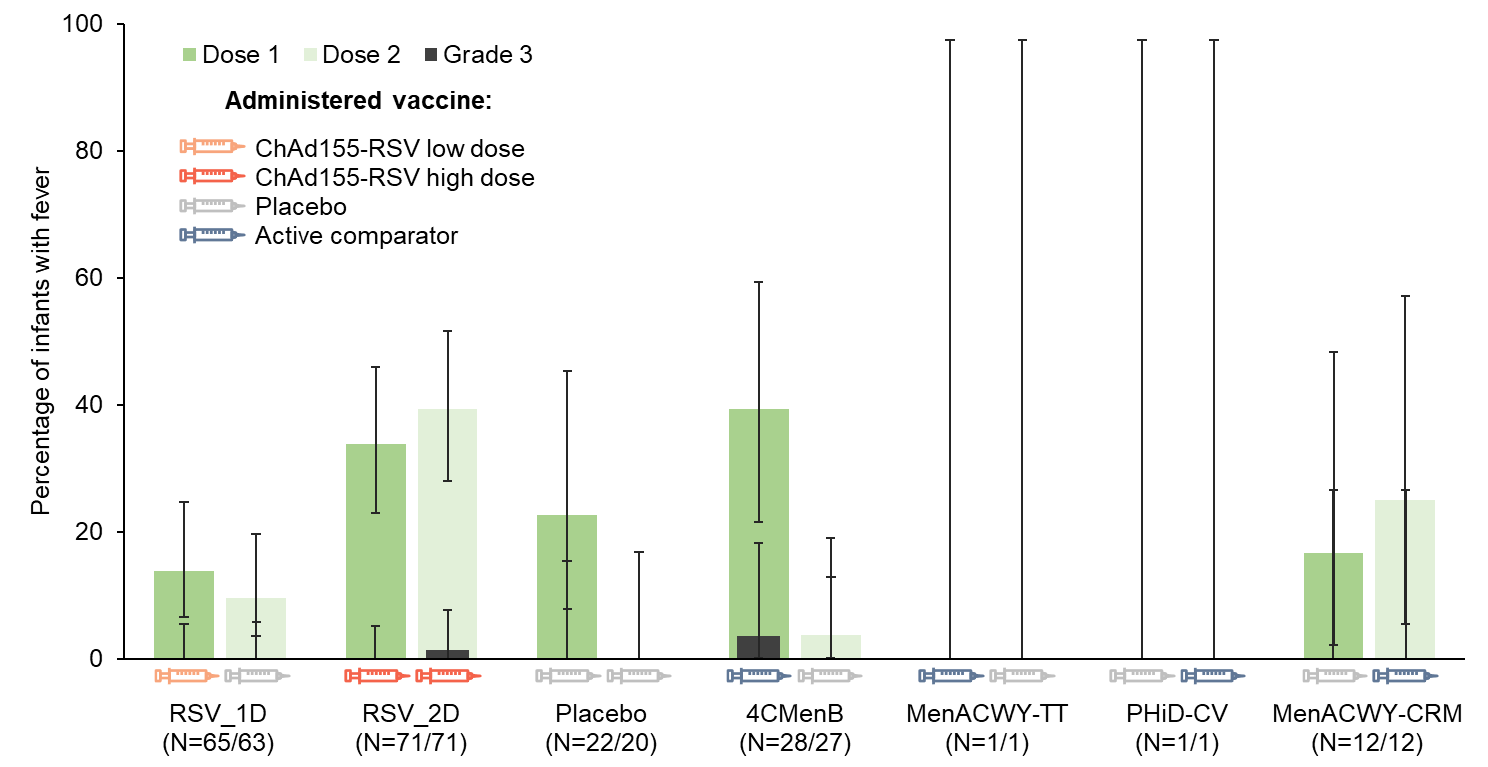


Error bars depict 95% confidence intervals. Fever was defined as a temperature ≥38.0°C; grade 3 fever as a temperature >40°C.

RSV, respiratory syncytial virus; RSV_1D, group receiving 1 low chimpanzee-derived replication-deficient adenoviral vector RSV vaccine (ChAd155-RSV) dose as dose 1 and placebo as dose 2; RSV_2D, group receiving 2 high ChAd155-RSV doses as dose 1 and 2; placebo, group receiving placebo as dose 1 and 2; 4CMenB, group receiving 4-component meningococcal serogroup B vaccine as dose 1 and placebo as dose 2; MenACWY-TT, group receiving meningococcal serogroups A, C, W, Y tetanus toxoid conjugate vaccine as dose 1 and placebo as dose 2; PHiD-CV, group receiving pneumococcal non-typeable *Haemophilus influenzae* protein D conjugate vaccine as dose 2 and placebo as dose 1; MenACWY-CRM, group receiving meningococcal serogroups A, C, W, Y CRM_197_ conjugate vaccine as dose 2 and placebo as dose 1; N, total number of participants with available data who received dose 1/dose 2.

**References**

1. Green CA, Scarselli E, Sande CJ, et al. Chimpanzee adenovirus- and MVA-vectored respiratory syncytial virus vaccine is safe and immunogenic in adults. Sci Transl Med **2015**; 7:300ra126.

2. Fulginiti VA, Eller JJ, Sieber OF, Joyner JW, Minamitani M, Meiklejohn G. Respiratory virus immunization. I. A field trial of two inactivated respiratory virus vaccines; an aqueous trivalent parainfluenza virus vaccine and an alum-precipitated respiratory syncytial virus vaccine. Am J Epidemiol **1969**; 89:435-48.

3. Kapikian AZ, Mitchell RH, Chanock RM, Shvedoff RA, Stewart CE. An epidemiologic study of altered clinical reactivity to respiratory syncytial (RS) virus infection in children previously vaccinated with an inactivated RS virus vaccine. Am J Epidemiol **1969**; 89:405-21.

4. Kim HW, Canchola JG, Brandt CD, et al. Respiratory syncytial virus disease in infants despite prior administration of antigenic inactivated vaccine. Am J Epidemiol **1969**; 89:422-34.

5. Chin J, Magoffin RL, Shearer LA, Schieble JH, Lennette EH. Field evaluation of a respiratory syncytial virus vaccine and a trivalent parainfluenza virus vaccine in a pediatric population. Am J Epidemiol **1969**; 89:449-63.

6. Dunn SR, Ryder AB, Tollefson SJ, Xu M, Saville BR, Williams JV. Seroepidemiologies of human metapneumovirus and respiratory syncytial virus in young children, determined with a new recombinant fusion protein enzyme-linked immunosorbent assay. Clin Vaccine Immunol **2013**; 20:1654-6.

7. Kutsaya A, Teros-Jaakkola T, Kakkola L, et al. Prospective clinical and serological follow-up in early childhood reveals a high rate of subclinical RSV infection and a relatively high reinfection rate within the first 3 years of life. Epidemiol Infect **2016**; 144:1622-33.

8. Modjarrad K, Giersing B, Kaslow DC, Smith PG, Moorthy VS, WHO RSV Vaccine Consultation Expert Group. WHO consultation on Respiratory Syncytial Virus Vaccine Development Report from a World Health Organization Meeting held on 23-24 March 2015. Vaccine **2016**; 34:190-7.
